# The effect of synthetic grass sports surfaces on the thermal environment: A systematic review

**DOI:** 10.1007/s00484-024-02679-5

**Published:** 2024-05-01

**Authors:** Gurpreet Singh, Benjamin Peterson, Ollie Jay, Christopher J. Stevens

**Affiliations:** 1 Physical Activity, Sport, and Exercise Research (PASER) Theme, Faculty of Health, Southern Cross University, Coffs Harbour, NSW Australia; 2 School of Health, Medical and Applied Sciences, Central Queensland University, Rockhampton, Australia; 3 Heat and Health Research Incubator, Faculty of Medicine and Health, University of Sydney, Camperdown, Australia

**Keywords:** Synthetic grass, Heat stress, Thermoregulation, Microclimate

## Abstract

There are concerns regarding high surface temperatures on synthetic grass sports surfaces influencing the surrounding thermal environment, potentially increasing heat stress and impacting athlete safety. As such, studies have investigated changes to the thermal environment surrounding synthetic grass surfaces in comparison to both natural grass, and synthetic surfaces with different features, but this body of research has not been systematically reviewed. Therefore, this systematic review aimed to (i) determine if there are differences in the thermal environment surrounding synthetic grass surfaces compared with natural grass surfaces, and (ii) determine if there are differences in the thermal environment between different types of synthetic grass surfaces. A systematic review adhering to the PRISMA guidelines was performed. The eligibility criteria required investigations to report at least one of the following environmental parameters on or directly above both a synthetic surface and a comparator group of either natural grass or an alternative synthetic grass surface used in sport: Air temperature, mean radiant temperature, humidity, wind velocity, unified heat stress indices (i.e. wet-bulb-globe temperature and heat index) and/or surface temperature. Twenty-three studies were identified*.* The only parameters that were consistently higher on synthetic grass compared to natural grass were the air temperature (range: 0.5–1.2 °C) and surface temperature (range: 9.4–33.7 °C), while the mean radiant temperature, humidity, wind velocity and wet-bulb-globe temperature remained similar or required more data to determine if any differences exist. Synthetic grass surfaces consisting of styrene butadiene rubber infill or a shock pad had increased surface temperatures, whereas surfaces with thermoplastic elastomer infill, Cool climate turf fibres or HydroChill had lower surface temperatures*.* This systematic review has demonstrated that air and surface temperatures can be increased on synthetic sports surfaces, compared to natural grass surfaces. However, it is uncertain whether the differences are enough to increase an individual’s heat stress risk and cause concern for athlete safety. While modifications to the turf infill or fibres can reduce synthetic surface temperatures, the effect of these features on the thermal environment as a whole is unclear. This review was prospectively registered with the Open Science Framework (Open Science Framework registration  10.17605/OSF.IO/BTKGE).

**Supplementary Information:**

The online version contains supplementary material available at 10.1007/s00484-024-02679-5.

## Introduction

Synthetic grass surfaces have become a popular alternative to natural grass for sports globally (Twomey et al. 2016). Traditionally, most sports are played on natural grass; however, to avoid extensive damage, natural grass surfaces require limits on the number of usage hours per week, which can also be severely reduced in wet-weather (Fleming 2011; Twomey et al. 2016). These usage restrictions, coupled with an increased demand for sports surfaces, have encouraged local governments to construct synthetic grass surfaces for sports that can withstand a greater number of playing hours while having other advantages, such as less ongoing maintenance and water consumption compared with natural grass surfaces (Fleming 2011; Jastifer et al. 2019). Synthetic grass surfaces also enable a faster speed of play in sports such as association football (soccer) and are aesthetically pleasing. Hence their wide adoption in sports, including soccer, the rugby codes and the Australian Football League.

Several synthetic grass surfaces have evolved to replicate natural grass surfaces (Twomey et al. 2016; Jastifer et al. 2019). First-generation synthetic grass surfaces were constructed directly onto a soil base with short nylon turf fibres (Claudio 2008), but athletes perceived increased risk of injury (Powell and Schootman 1992) as the surfaces were considered too ‘stiff’ to compete on (Claudio 2008). Second-generation synthetic grass surfaces were developed to address these issues, containing longer polypropylene turf fibres (20–35 mm), a shock pad, and silica sand infill spread across the turf fibres to keep them upright (Jastifer et al. 2019). However, the silica sand infill caused friction burns and damaged the turf fibres. Modern synthetic grass surfaces (i.e. third and fourth-generation) were introduced to reduce this friction, and these surfaces have the option of several infill variations, including styrene-butadiene rubber (SBR), thermoplastic elastomer (TPE), ethylene-propylene-diene monomer (EPDM) or cork infill. Third and fourth-generation surfaces also have longer polyethylene-polypropylene turf fibres (50–60 mm) and can contain Cool climate turf fibres or a HydroChill product to reduce heat, which, when coupled with contemporary infill options, increases shock absorption and surface traction and allows athletes to wear studded shoes (Severn et al. 2011). Thus, the overall effect of these synthetic grass attributes results in very similar playing conditions to natural grass (Jastifer et al. 2019). Accordingly, influential governing bodies in sport (i.e. FIFA, World Rugby, National Rugby League and the Australian Football League) stipulate that only third and fourth-generation synthetic grass surfaces are approved for their competitions.

Research has been conducted into the effect of synthetic grass surfaces on the thermal environment and concerns have been raised regarding high surface temperatures on synthetic grass sports surfaces (Buskirk et al. 1971; Claudio 2008; McNitt et al. 2008; Twomey et al. 2016; Jim 2016; Abraham 2019). Specifically, these studies suggested that heat stress remains a concern on synthetic grass surfaces, which is a potential risk to athlete safety (Jim 2016; Jastifer et al. 2019). An individual’s core temperature increases when internal heat production (i.e. metabolic processes) exceeds the body’s ability to dissipate heat from the skin surface into the environment via convection, radiation, conduction and evaporation (Cramer and Jay 2016). However, the heat loss capacity depends on the temperature gradient between the body surface and the environment, which is governed by four environmental parameters, including the air temperature, mean radiant temperature, absolute humidity, and wind velocity. If the air and radiant temperature are hotter than the skin temperature, heat loss is hindered and can lead to heat gain (Cramer and Jay 2016). It remains unknown whether synthetic grass surfaces influence these key environmental parameters.

While studies have investigated synthetic grass surface temperatures and their effects on the thermal environment, this research has yet to be systematically reviewed; therefore, it is difficult for scientists, facility managers and policymakers to access and interpret. An evidence-based resource is required to inform local governments and governing sports bodies about potential environmental risks that result from physical activity, exercise and sport on synthetic grass surfaces. Therefore, this review aims to (i) determine if there are differences in the thermal environment surrounding synthetic grass surfaces compared with natural grass surfaces, and (ii) determine if there are differences in the thermal environment between different types of synthetic grass surfaces.

## Methods

### Protocol

This systematic review followed the Preferred Reporting Items for Systematic Reviews and Meta-Analyses (PRISMA) statement (Page et al. 2021) and was prospectively registered with the Open Science Framework (Open Science Framework registration  10.17605/OSF.IO/BTKGE). Modifications to the original registration were made such as not including a meta-analysis due to the availability of data, a more detailed inclusion criterion and the aims were adjusted by defining the ‘thermal environment’ using the following sentence “air temperature, mean radiant temperature, humidity, wind velocity, unified heat stress indices (i.e. wet-bulb-globe temperature and heat index) and surface temperature)”. These changes did not impact the findings of the review.

### Eligibility criteria

To meet the eligibility criteria, studies were required to: (i) contain original research; (ii) be peer-reviewed; (iii) be published in English; (iv) and have reported at least one of the following environmental parameters on or directly above both a synthetic surface and a comparator group of either natural grass or an alternative synthetic grass surface used in sport: surface temperature, air temperature, radiant temperature, humidity, solar radiation, wet-bulb-globe temperature or wind velocity. All synthetic grass generations were included (i.e. first-, second, third-, and fourth-generation). Grey literature, including conference proceedings, dissertations, and editorials were not eligible for inclusion.

### Information sources and search strategy

An electronic database search was conducted in Academic Search Premier, Medline, SPORTdiscus with Full Text, Sage Journals, Web of Science (Core Collection), Scopus, and Proquest from inception until 6th April, 2023. No limits were applied to the publication year, as such, searches were conducted without any specified date range up to the 6^th^April, 2023*.* The search string used to retrieve relevant articles was; (“synthetic grass” OR “synthetic turf” OR “artificial grass” OR “artificial turf” OR “synthetic surface” OR “artificial surface” OR “astroturf” OR “green synthetic turfgrass” OR “synthetic sport* surface*”) AND (temperature* OR microclimat* OR humid* OR radiation OR radiant OR vapour OR ultraviolet). A manual search of the reference lists of included studies was performed, resulting in no new studies.

### Screening process

The titles and abstracts of all studies retrieved through database searches were independently screened as well as the full texts. Reasons for exclusion were documented (see Supplementary file 1), and any conflicts were resolved through critical discussions between authors. The authors of the original studies were contacted if further data were required.

### Quality assessment

A critical appraisal tool for observational studies (i.e. Health Evidence Bulletins - Wales: Questions to assist with the critical appraisal of an observational study e.g. cohort, case-control, cross-sectional; Weightman et al. 2004) was used to assess the quality of the studies. This tool was developed to assess the quality of observational studies, including cohort, case-control and cross-sectional designs (Weightman et al. 2004). Given that the studies included in this review predominately used observational study designs, this quality assessment tool was deemed appropriate. Importantly, this tool is descriptive without calculation of an overall quality score.

### Data extraction and analysis

The following data was extracted: authors, publication date, country, surface types, environmental parameters (i.e. surface temperature, air temperature, radiant temperature, humidity, wet-bulb-globe temperature, wind velocity and solar radiation), environmental conditions, measurement device(s) and the height of the device(s) when used directly above a surface, duration of data collection and season. Study results were summarised through narrative synthesis, and the findings of individual studies were presented in Table 1. Based on data extraction, a meta-analysis was not performed due to methodological heterogeneity between studies, under-reporting of data required for pooled calculation of mean differences, and concern about the quality and appropriateness of reporting of data within individual studies, which are explained further within the ‘quality appraisal’ section of the results. It has previously been recommended that meta-analyses are likely to produce an inappropriate summary in the presence of publication and/or reporting bias (Higgins et al. 2023).

**Table 1:** A summary of the results reported in each study

Study	Results (data presented as mean ± SD, unless stated otherwise)
(Bozdogan Sert et al. 2021)	Surface temperature (absolute): Synthetic grass = 66.7 °C (SD NR) vs. Natural grass = 36.5 °C & 36.4 °C (*p* value NR)
(Carvalho et al. 2021)	Surface temperature (maximum): Synthetic grass plot = 61.0 °C (SD NR) vs. Natural grass plot = 41.0 °C (SD NR) and (*p* value NR)
(Grundstein and Cooper 2020)	Tg (median): 1.42–1.53 °C higher over synthetic than natural WBGT morning (median): data NR *(p* = 0.062) WBGT midday (median): data NR *(p* = 0.776) Synthetic and natural grass afternoon median WBGT: (data NR) *(p* = 0.437)
(Guyer et al. 2021)	Surface temperature June (range): Synthetic = 24.3–77.9 °C; Natural = 23.2–38.4 °C Ta June (mean ± SD) & max: Synthetic = 36.4 °C ± 3.7 °C & 47.6 °C; Natural = 35.9 °C ± 3.1 °C & 47.6 °C RH June (mean ± SD): Synthetic = 12% ± 7.4%; Natural = 13% ± 4.0% Wind velocity June (mean ± SD): Synthetic = 1.2 m^**.**^s^−1^ ± 0.8 m^**.**^s^−1^; Natural = 1.3 m^**.**^s^−1^ ± 0.9 m^**.**^s^−1^ Surface temperature August (range): Synthetic = 25.2–68.1 °C; Natural = 24.5–41.5 °C Ta August (mean ± SD) & max: Synthetic = 39.1 °C ± 3.9 °C & 47.9 °C; Natural = 38.1 °C ± 3.2 °C & 49.7 °C RH August (mean ± SD): 21% ± 12.4%; Natural = 22% ± 9.9% Wind velocity August (mean ± SD): Synthetic = 1.1 m^**.**^s^−1^ ± 0.8 m/s^−1^; Natural = 1.3 m^**.**^s^−1^ ± 1.0 m^**.**^s^−1^
(Hardin and Vanos 2018)	Ta (mean, SD NR): Synthetic grass = 37.0 °C; Natural = 38.8 °C; Moist natural grass = 32.6 °C Wind velocity (mean, SD NR): Synthetic grass = 3.25 m^**.**^s^−1^; Natural grass = 1.04 m^**.**^s^−1^; Moist natural grass = 0.83 m^**.**^s^−1^ Horizontal short- and long-wave radiation (mean, SD NR): Synthetic grass = 535.3 W.m^−2^; Natural grass = 547.9 W.m^−2^; Moist natural grass = 446.2 W.m^−2^
(Jim 2016)	Surface temperature (maximum): Synthetic grass day 1 & 2 = 70.2 °C & 74.6 °C; Natural grass = 38.1 °C & 36.9 °C Surface temperature (minimum): Synthetic grass day 1 & 2 = 23.1 °C & 23.4 °C; Natural grass = 23.1 °C & 23.1 °C Ta (maximum): Synthetic grass day 1 & 2150 cm = 35.4 °C & 36.0 °C; 50 cm day 1 & 2 = 36.6 °C & 37.1 °C; 15 cm day 1 & 2 = 42.7 °C & 54.2 °C Ta (minimum): Synthetic grass day 1 & 2150 cm = 28.6 °C & 29.1 °C; 50 cm day 1 & 2 = 28.4 °C & 28.8 °C; 15 cm day 1 & 2 = 28.0 °C & 28.4 °C Ta (maximum): Natural grass day 1 & 2150 cm = 34.4 °C & 34.8 °C; 15 cm day 1 & 2 = 34.4 °C & 35.0 °C; 15 cm day 1 & 2 = 37.5 °C & 36.9 °C Ta (minimum): Natural grass day 1 & 2150 cm = 28.6 °C & 28.9 °C; 50 cm day 1 & 2 = 28.5 °C & 28.7 °C; 15 cm day 1 & 2 = 28.2 °C & 28.3 °C Reflected solar (maximum): Synthetic grass day 1 & day = 47.3 W.m^−2^ & 48.9 W.m^−2^; Natural grass = 192.0 W.m^−2^ & 157.8 W.m^−2^ Ground thermal radiation (maximum): Synthetic day 1 & 2 = 714.3 W.m^−2^ & 743.8 W.m^−^ 2; Natural grass = 548.1 & 541.5 W.m^−2^ Albedo (maximum): Synthetic day 1 & 2 = 0.15 & 0.14; Natural grass = 0.33 & 0.29 Albedo (minimum): Synthetic day 1 & 2 = 0.05 & 0.05; Natural grass = 0.19 & 0.16
(Jim 2017)	Surface temperature (maximum): Synthetic grass day 1, 2 & 3 = 73.8 °C, 65.8 °C, 52.5 °C; Natural grass = 37.3 °C, 36.1 °C, 31.6 °C Surface temperature (minimum): Synthetic grass day 1, 2 & 3 = 23.2 °C, 22.8 °C, 23.1 °C; Natural grass = 23.2 °C, 22.0 °C, 22.7 °C Ta (maximum): Synthetic grass day 1, 2 & 3150 cm = 34.4 °C, 33.6 °C, 32.4 °C; Minimum = 29.0 °C, 28.2 °C, 28.1 °C Ta (maximum): Synthetic grass day 1, 2 & 3 50 cm = 36.9 °C, 34.7 °C, 33.5 °C; Minimum = 28.7 °C, 27.9 °C, 27.9 °C Ta (maximum): Synthetic grass day 1, 2 & 3 15 cm = 44.1 °C, 38.4 °C, 37.8 °C; Minimum = 28.2 °C, 27.6 °C, 37.4 °C Ta (maximum): Natural grass day 1, 2 & 3150 cm = 34.4 °C 32.3 °C, 30.5 °C; Minimum = 28.3 °C, 28.2 °C, 27.7 °C Ta (maximum): Natural grass day 1, 2 & 3 50 cm = 34.6 °C, 32.4 °C, 31.0 °C; Minimum = 28.8 °C, 28.8 °C, 27.9 °C Ta (maximum): Natural grass day 1, 2 & 3 15 cm = 37.6 °C, 34.7 °C, 32.4 °C; Minimum = 28.3 °C, 27.5 °C, 28.1 °C
(Kandelin et al. 1976)	Surface temperature (mean SD NR): Synthetic = 49.3 °C vs. Natural = 39.9 °C; *p* < 0.01 Ta at 0.9 m (mean SD NR): Synthetic = 32.7 °C vs. Natural = 31.3 °C; *p* < 0.01 Ta at 1.5 m (mean SD NR): Synthetic = 32.3 °C vs. Natural = 31.1 °C; *p* < 0.01 Subsurface temperature (mean daily high SD NR): Synthetic = 42.4 °C; Natural grass = 28.8 °C Subsurface (maximum): Synthetic = 46.2 °C; Natural grass = 30.0 °C
(Liu and Jim 2021)	Surface temperature: Synthetic grass sunny day; mean = 48.1 °C SD NR, min = 29.2 °C, max = 65.3 °C Surface temperature: Synthetic grass cloudy day; mean = 38.1 °C SD NR, min = 26.5 °C, max = 59.9 °C Surface temperature: Synthetic grass overcast day; mean = 32.5 °C SD NR; min = 28.3 °C, max = 47.8 °C Surface temperature: Natural grass sunny day; mean = 35.2 °C SD NR, min = 29.1 °C, max = 40.4 °C Surface temperature: Natural grass cloudy day; mean = 32.2 °C SD NR, min = 26.3 °C, max = 38.9 °C Surface temperature: Natural grass overcast day; mean = 30.0 °C SD NR, min = 28.8 °C, max = 34.1 °C Ta: Synthetic grass sunny day; mean = 32.5 °C SD NR, min = 28.3 °C, max = 34.8 °C; Natural grass; mean = 31.9 °C SD NR, min = 28.3 °C, max = 34.8 °C Ta: Synthetic grass cloudy day; mean = 30.3 °C SD NR, min = 26.0 °C, max = 33.6 °C; Natural grass; mean = 30.0 °C SD NR, min = 26.0 °C, max = 32.3 °C Ta: Synthetic grass overcast day; mean = 29.4 °C SD NR, min = 28.1 °C, max = 32.4 °C; Natural grass; mean = 28.8 °C SD NR, min = 27.7 °C, max = 30.1 °C RH: Synthetic grass sunny day; mean = 72.2% SD NR, min = 58.4%, max = 91.4%; Natural grass; mean = 76.1% SD NR, min = 66.6%, max = 61.9% RH: Synthetic grass cloudy day; mean = 82.6% SD NR, min = 65.4%, max = 98.2%; Natural grass; mean = 84.8% SD NR, min = 73.2%, max = 98.8% RH: Synthetic grass overcast day; mean = 81.9% SD NR, min = 73.0%, max = 88.3%; Natural grass; mean = 85.6% SD NR, min = 78.4%, max = 92.2% Wind velocity: Synthetic grass sunny day; mean = 0.47 m^**.**^s^−1^ SD NR, min = 0.00 m^**.**^s^−1^, max = 1.30 m^**.**^s^−1^; Natural grass; mean = 0.47 m^**.**^s^−1 ^SD NR, min = 0.00 m^**.**^s^−1^, max = 1.30 m^**.**^s^−1^ Wind velocity: Synthetic grass cloudy day; mean = 0.37 m^**.**^s^−1^ SD NR, min = 0.00 m^**.**^s^−1^, max = 1.48 m^**.**^s^−1^; Natural grass; mean = 0.37 m^**.**^s^−1^ SD NR, min = 0.00 m^**.**^s^−1^, max = 1.48 m^**.**^s^−1^ Wind velocity: Synthetic grass cloudy day; mean = 0.78 m^**.**^s^−1^ SD NR, min = 0.19 m^**.**^s^−1^, max = 1.67 m^**.**^s^−1^; Natural grass; mean = 0.78 m^**.**^s^−1^ SD NR, min = 10.19 m^**.**^s^−1^, max = 1.67 m^**.**^s^−1^ Tw: Synthetic grass sunny day; mean = 28.15 °C SD NR, min = 26.30 °C, max = 29.90 °C; Natural grass; mean = 28.24 °C SD NR, min = 26.20 °C, max = 29.90 °C Tw: Synthetic grass cloudy day; mean = 27.65 °C SD NR, min = 25.90 °C, max = 29.80 °C; Natural grass; mean = 27.51 °C SD NR, min = 25.40 °C, max = 29.50 °C Tw: Synthetic grass overcast day; mean = 26.58 °C SD NR, min = 25.30 °C, max = 28.00 °C; Natural grass; mean = 26.63 °C SD NR, min = 25.40 °C, max = 27.80 °C Tg: Synthetic grass sunny day; mean = 41.59 °C SD NR, min = 28.70 °C, max = 53.50 °C; Natural grass; mean = 42.26 °C SD NR, min = 28.60 °C, max = 53.20 °C) Tg: Synthetic grass cloudy day; mean = 35.29 °C SD NR, min = 26.10 °C, max = 48.70 °C; Natural grass; mean = 36.04 °C SD NR, min = 25.50 °C, max = 50.80 °C Tg: Synthetic grass overcast day; mean = 31.50 °C SD NR, min = 27.20 °C, max = 42.90 °C; Natural grass; mean = 31.20 °C SD NR, min = 27.40 °C, max = 43.40 °C WBGT: Synthetic grass sunny day; mean = 31.27 °C SD NR, min = 27.50 °C, max = 34.81 °C; Natural grass; mean = 31.42 °C SD NR, min = 27.31 °C, max = 34.39 °C WBGT: Synthetic grass cloudy day; mean = 29.44 °C SD NR, min = 25.95 °C, max = 33.80 °C; Natural grass; mean = 29.47 °C SD NR, min = 25.48 °C, max = 34.04 °C WBGT: Synthetic grass overcast day; mean = 27.84 °C SD NR, min = 26.03 °C, max = 31.42 °C; Natural grass; mean = 27.77 °C SD NR, min = 26.22 °C, max = 31.14 °C Shortwave upwards radiation: Synthetic grass sunny day; mean = 29.37 W.m^−2^ SD NR, min = 5.71 W.m^−2^, max = 49.7 W.m^−2^ Shortwave upwards radiation: Synthetic grass cloudy day; mean = 18.14 W.m^−2^ SD NR, min = 3.26 W.m^−2^, max = 54.62 W.m^−2^ Shortwave upwards radiation: Synthetic grass overcast day; mean = 10.26 W.m^−2^ SD NR, min = 2.46 W.m^−2^, max = 32.62 W.m^−2^ Shortwave upwards radiation: Natural grass sunny day; mean = 99.93 W.m^−2^ SD NR, min = 7.60 W.m^−2^, max = 191.97 W.m^−2^ Shortwave upwards radiation: Natural grass cloudy day; mean = 62.56 W.m^−2^ SD NR, min = 3.80 W.m^−2^, max = 212.89 W.m^−2^ Shortwave upwards radiation: Natural grass overcast day; mean = 29.27 W.m^−2^ SD NR, min = 6.66 W.m^−2^, max = 106.41 W.m^−2^ Longwave upwards radiation: Synthetic grass sunny day; mean = 608.66 W.m^−2^ SD NR, min = 473.50 W.m^−2^, max = 743.79 W.m^−2^ Longwave upwards radiation: Synthetic grass cloudy day; mean = 533.95 W.m^−2^ SD NR, min = 457.35 W.m^−2^, max = 697.67 W.m^−2^ Longwave upwards radiation: Synthetic grass overcast day; mean = 495.01 W.m^−2^ SD NR, min = 468.10 W.m^−2^, max = 601.39 W.m^−2^ Longwave upwards radiation: Natural grass sunny day; mean = 513.26 W.m^−2^ SD NR, min = 472.97 W.m^−2^, max = 548.13 W.m^−2^ Longwave upwards radiation: Natural grass cloudy day; mean = 492.97 W.m^−2^ SD NR, min = 455.94 W.m^−2^, max = 537.76 W.m^−2^ Longwave upwards radiation: Natural grass overcast day; mean = 479.00 W.m^−2^ SD NR, min = 471.08 W.m^−2^, max = 504.98 W.m^−2^ Heat index: Synthetic grass sunny day; mean = 42.53 °C SD NR, min = 48.45 °C, max = 35.04 °C; Natural grass; mean = 40.49 °C SD NR, min = 50.83 °C, max = 34.84 °C Heat index: Synthetic grass cloudy day; mean = 39.13 °C SD NR, min = 47.89 °C, max = 28.40 °C; Natural grass; mean = 39.03 °C SD NR, min = 47.34 °C, max = 28.33 °C Heat index: Synthetic grass overcast day; mean = 36.30 °C SD NR, min = 43.24 °C, max = 32.49 °C; Natural grass; mean = 35.49 °C SD NR, min = 39.51 °C, max = 32.00 °C
(Loveday et al. 2019a)	Apparent temperature (min & max) winter 2016: Synthetic = − 5.1-34.0 °C Apparent temperature (min & max) winter 2016: Natural = − 2.3-21.9 °C Apparent temperature (min & max) spring 2016: Synthetic = − 2.3-55.5 °C Apparent temperature (min & max) spring 2016: Natural = 1.2–30.4 °C Apparent temperature (min & max) summer 2018: Synthetic = 12.0–75 °C Apparent temperature (min & max) summer 2018: Natural = 13.5–44.8 °C Apparent temperature (min & max) autumn 2017: Synthetic = 3.9–58.3 °C Apparent temperature (min & max) autumn 2017: Natural = 4.2–38.8 °C Apparent temperature (min & max) summer 2019: Synthetic = 16.0–73.9 °C Apparent temperature (min & max) summer 2019: Natural = 15.5–48.5 °C
(Loveday et al. 2019b)	Albedo: Synthetic grass: 0.10, 0.12, 0.11; Natural grass: 0.20, 0.22, 0.22
(McNitt et al. 2008)	Surface temperature (absolute): Astroturf day 1, 2 & 3 = 51.9 °C,52.4 °C & 53.8 °C Surface temperature (absolute): Astroplay day 1, 2 & 3 = 46.8 °C,51.9 °C & 59.5 °C Surface temperature (absolute): Experimental turf day 1, 2 & 3 = 48.4 °C, 52.3 °C & 58.4 °C Surface temperature (absolute): Field turf day 1, 2 & 3 = 46.8 °C, 58.1 °C & 64.8 °C Surface temperature (absolute): Geoturf day 1, 2 & 3 = 53.1 °C,61.1 °C & 70.8 °C Surface temperature (absolute): Nexturf day 1, 2 & 3 = 51.1 °C, 56.4 °C & 71.5 °C Surface temperature (absolute): Omnigrass 41 day 1, 2 & 3 = 48.1 °C, 53.2 °C & 64.2 °C Surface temperature (absolute): Omnigrass 51 day 1, 2 & 3 = 49.2 °C, 55.6 °C & 63.1 °C Surface temperature (absolute): Sofsport day 1, 2 & 3 = 49.9 °C, 54.6 °C & 62.6 °C Surface temperature (absolute): Sprinturf day 1, 2 & 3 = 45.4 °C, 48.1 °C & 54.4 °C Ta (absolute): Asroturf day 1, 2 & 3 = 25.9 °C 25.5 °C & 28.9 °C Ta (absolute): Astroplay day 1, 2 & 3 = 25.6 °C, 25.7 °C & 30.5 °C Ta (absolute): Experimental turf day 1, 2 & 3 = 24.8 °C, 26.1 °C & 30.8 °C Ta (absolute): Field turf day 1, 2 & 3 = 26.1 °C, 25.6 °C & 28.3 °C Ta (absolute): Geoturf day 1, 2 & 3 = 26.5 °C, 25.9 °C & 29.5 °C Ta (absolute): Nexturf day 1, 2 & 3 = 25.1 °C, 25.1 °C & 30.6 °C Ta (absolute): Omnigrass 41 day 1, 2 & 3 = 25.9 °C, 25.8 °C & 29.3 °C Ta (absolute): Omnigrass 51 day 1, 2 & 3 = 26.3 °C, 25.6 °C & 29.4 °C Ta (absolute): Sofsport 51 day 1, 2 & 3 = 27.0 °C, 25.5 °C & 29.1 °C Ta (absolute): Sprinturf: day 1, 2 & 3 = 26.4 °C, 25.8 °C & 30.0 °C
(Petrass et al. 2014a)	Surface temperature (Mean SD NR): Synthetic plot with SBR infill = 53.5 °C vs. Synthetic plot with TPE infill = 45.6 °C; *p* < 0.001 Surface temperature (mean SD NR): Synthetic plot with a shock pad = 50.3 °C vs. Synthetic plot without a shock pad = 48.5 °C; *p =* 0.001 Surface temperature (mean SD NR): Synthetic grass plot with sand/organic infill = 48.1 °C
(Petrass et al. 2014b)	Surface temperature (mean ± SD): Third-generation synthetic grass with CoolClimate turf = 40.06 °C ± 12.7 °C vs. natural grass = 27.6 °C ± 7.4 °C; *p* < 0.001 Surface temperature (mean ± SD): Third-generation synthetic grass = 46.0 °C ± 14.4 °C vs natural grass = 23.8 °C ± 5.3 °C; *p* < 0.001 Surface temperature (range): Third-generation synthetic with CoolClimate synthetic = 19.5 °C–71.1 °C Surface temperature (range): Third-generation synthetic = 40.4 °C–80.1 °C Surface temperature (range): Natural grass metro = 15.2–53.2 °C Surface temperature (range): Natural grass regional = 11.8–36.0 °C Ta (mean ± SD) & (range): Third-generation synthetic with CoolClimate synthetic = 25.5 °C ± 4.1 °C (18.5–35.1 °C) Ta (mean ± SD) & (range): Third-generation synthetic = 25.7 °C ± 5.8 °C (14.78–35.8 °C) Ta (mean ± SD) & (range): Natural grass metro = 24.7 °C ± 4.1 °C (18.1–34.8 °C) Ta (mean ± SD) & (range): Natural grass regional = 25.4 °C ± 6.0 °C (14.4–36.1 °C) RH (mean ± SD) & (range): Third-generation synthetic with CoolClimate synthetic = 50.96% ± 12.78% (18.2–73.8%) RH (mean ± SD) & (range): Third-generation synthetic = 40.26% ± 11.35% (17.6–67.6%) RH (mean ± SD) & (range): Natural grass metro = 53.67% ± 11.2% (31.5–73.7%) RH (mean ± SD) & (range): Natural grass regional = 43.42% ± 12.23% (18.7–67.7%) Wind velocity (mean ± SD) & (range): Third-generation synthetic with CoolClimate synthetic = 7.65 km/h ± 6.49 km/h (0–34.4 km/h) Wind velocity (mean ± SD) & (range): Third-generation synthetic = 8.28 km/h ± 5.49 km/h (0–30.0 km/h) Wind velocity (mean ± SD) & (range): Natural grass metro = 8.10 km/h ± 6.39 km/h (0–22.5 km/h) Wind velocity (mean ± SD) & (range): Natural grass regional = 7.43 km/h ± 4.19 km/h (0–18.5 km/h) Tw (mean ± SD) & (range): Third-generation synthetic with CoolClimate synthetic = 18.29 °C ± 5.10 °C (12.0–39.8 °C) Tw (mean ± SD) & (range): Third-generation synthetic = 17.29 °C ± 4.08 °C (10.2–34.5 °C) Tw (mean ± SD) & (range): Natural grass metro = 17.22 °C ± 3.89 °C (12.6–36.3 °C) Tw (mean ± SD) & (range): Natural grass regional = 17.00 °C ± 3.71 °C (9.4–23.2 °C)
(Pfautsch et al. 2022)	Surface temperature (Mean ± SD): Synthetic grass (30 mm fibres) in the sun = 57.5 °C ± 14.4 °C Surface temperature (Mean ± SD): Synthetic grass (30 mm fibres) in the shade = 32.2 °C ± 7.7 °C Surface temperature (Mean ± SD): Synthetic grass (40 mm fibres) in the sun = 56.6 °C ± 13.6 °C Surface temperature (Mean ± SD): Synthetic grass (40 mm fibres) in the shade = 32.5 °C ± 7.5 °C Surface temperature (Mean ± SD): Synthetic grass (13 mm red fibres) in the sun = 58.2 °C ± 14.5 °C Surface temperature (Mean ± SD): Synthetic grass (13 mm red fibres) in the shade = 31.9 °C ± 7.7 °C Surface temperature (Mean ± SD): Synthetic grass (13 mm green fibres) in the sun = 56.6 °C ± 14.3 °C Surface temperature (Mean ± SD): Synthetic grass (13 mm green fibres) in the shade = 32.6 °C ± 7.3 °C Surface temperature (maximum): Synthetic grass (30 mm fibres) in the sun = 80.1 °C Surface temperature (maximum): Synthetic grass (30 mm fibres) in the shade = 42.1 °C Surface temperature (maximum): Synthetic grass (40 mm fibres) in the sun = 84.5 °C Surface temperature (maximum): Synthetic grass (40 mm fibres) in the shade = 41.7 °C Surface temperature (maximum): Synthetic grass (13 mm red fibres) in the sun = 77.7 °C Surface temperature (maximum): Synthetic grass (13 mm red fibres) in the shade = 41.6 °C Surface temperature (maximum): Synthetic grass (13 mm green fibres) in the sun = 74.3 °C Surface temperature (maximum): Synthetic grass (13 mm green fibres) in the shade = 42.6 °C Surface temperature (minimum): Synthetic grass (30 mm fibres) in the sun = 20.2 °C Surface temperature (minimum): Synthetic grass (30 mm fibres) in the shade = 15.5 °C Surface temperature (minimum): Synthetic grass (40 mm fibres) in the sun = 20.3 °C Surface temperature (minimum): Synthetic grass (40 mm fibres) in the shade = 15.4 °C Surface temperature (minimum): Synthetic grass (13 mm red fibres) in the sun = 21.0 °C Surface temperature (minimum): Synthetic grass (13 mm red fibres) in the shade = 16.0 °C Surface temperature (minimum): Synthetic grass (13 mm green fibres) in the sun = 18.0 °C Surface temperature (minimum): Synthetic grass (13 mm green fibres) in the sun = 14.9 °C Surface temperature (Mean ± SD): Natural grass in the sun = 32.2 °C ± 5.6 °C Surface temperature (Mean ± SD): Natural grass in the shade = 26.1 °C ± 4.4 °C Maximum surface temperature: Natural grass in the sun = 55.1 °C Minimum surface temperature: Natural grass in the sun = 34.0 °C
(Pryor et al. 2017)	WBGT (mean difference ± SD): Synthetic grass vs. natural grass = 0.09 °C ± 2.33 °C; *p* > 0.05 WBGT (mean difference ± SD): Astroturf vs. natural grass = 0.61 °C ± 1.88 °C; *p* > 0.05 WBGT (mean difference ± SD; range): Between natural grass and synthetic turfgrass = 0.09 °C ± 2.33 °C; (− 3.50 °C–4.05 °C) WBGT (mean difference ± SD; range): Between natural grass and Astroturf = 0.61 °C ± 1.88 °C; (− 2.25 °C–3.45 °C) WBGT (mean difference ± SD; range) Between third-generation synthetic grass difference and Astroturf = 0.52 °C ± 2.27 °C; (− 3.65 °C–5.20 °C)
(Ramsey 1982)	Td (mean difference ± SD): Between synthetic and natural grass = 1.7 °C ± 1.2 °C (*p* value NR) WBGT (mean difference ± SD): Between synthetic and natural grass = 0.5 °C ± 0.8 °C (*p* value NR) Tg (mean difference ± SD): Between synthetic and natural grass = 2.0 °C ± 1.9 °C (*p* value NR) Tw (mean difference ± SD): Between synthetic and natural grass = − 0.3 °C ± 0.8 °C (*p* value NR)
Shi and Jim 2022)	Surface temperature (maximum): Synthetic grass surface temperature in sunny conditions = 74.6 °C Surface temperature (maximum): Synthetic grass surface temperature in cloudy conditions = 52.5 °C Surface temperature difference: Between synthetic and natural grass = 4.6–18.3 °C; *p < 0.05* Ta difference: Between synthetic and natural grass (data not available) = *p* < 0.05 Mean radiant temperature difference: Between synthetic and natural grass in sunny conditions = 3.4 °C; *p* < 0.05 Mean radiant temperature difference: Between synthetic and natural grass in cloudy conditions = 0.8 °C; *p* < 0.05 Mean radiant temperature difference: Between synthetic and natural grass in overcast conditions = 0.4 °C; *p* < 0.05
(Thoms et al. 2014)	Surface temperature (range): Synthetic grass = − 9.8 °C-86.4 °C
(Twomey et al. 2016)	Surface temperature (Mean ± SD): Third-generation synthetic = 46.3 °C ± 15.6 °C vs.35.1 °C ± 10.0 °C; *p* < 0.001 Surface temperature (Mean ± SD): Artificial Street soccer field = 38.8 °C ± 10.4 °C Surface temperature (min & max): Third-generation synthetic grass = 18.7 °C & 86.6 °C Surface temperature (min & max): Natural grass = 3.9 °C & 65.1 °C Surface temperature (min & max): Artificial Street soccer field =17.5 °C & 64.1 °C
(Villacañas et al. 2017)	Surface temperature (Mean ± SD): Synthetic grass with SBR infill = 61.2 ± 6.5 °C vs. Synthetic grass with TPE infill = 58.0 ± 5.0 °C; *p* < 0.001 Surface temperature (Mean ± SD): Synthetic grass with monofilament = 57.9 °C ± 6.2 °C vs. Synthetic with fibrillated fibres = 61.9 °C ± 6.1 °C; *p* = 0.0653 Surface temperature (Mean ± SD): Synthetic field age < 5 years = 60.7 °C ± 6.3 °C vs. Synthetic field age > 5 years = 58.7 °C ± 6.1 °C; *p* = 0.393 Surface temperature (Mean ± SD): Synthetic field use < 35 hours/week = 60.9 °C ± 6.6 °C vs. Synthetic field use > 35 hour/week =. 58.8 °C ± 5.4 °C; *p* = 0.529
(Wardenaar et al. 2022)	Surface temperature (Mean ± SD): Synthetic grass = 35.2 °C ± 1.1 °C vs. Natural grass = 32.4 °C ± 0.9 °C; *p* < 0.05 Globe temperature (Mean ± SD): Synthetic grass = 48.3 °C ± 0.9 °C vs. Natural grass = 45.5 °C ± 1.0 °C; *p* < 0.05 WBGT (Mean ± SD): Synthetic grass = 27.5 °C ± 0.4 °C vs. Natural grass = 25.3 °C ± 0.4 °C; *p* < 0.05 Wind velocity (Mean ± SD): Synthetic grass = 2.8 m^**.**^s^−1^ ± 1.57 m^**.**^s^−1^vs. Natural grass = 1.5 m^**.**^s^−1^ ± 1.01 m^**.**^s^−1^ *p* < 0.05 Outgoing longwave radiation (Mean ± SD): Synthetic grass = 551 W.m^−2^ ± 7.1 W.m^−2^ vs. Natural grass = 514 W/m^−2^ ± 3.3 W.m^−2^ Outgoing solar radiation (Mean ± SD): Natural grass = 113 W.m^−2^ ± 3.0 W.m^−2^ vs. Synthetic grass = 58 W.m^−2^ ± 1.2 W.m^−2^; *p* < 0.05 Albedo: Natural grass = 12.6% vs. Synthetic grass = 6.9%; *p* < 0.05 Relative humidity (Mean ± SD): Synthetic grass = 15% ± 1.6% vs. Natural grass = 13.1% ± 1.2%; *p* > 0.05 Surface temperature (mean ± SD): Synthetic grass with HydroChill = 67.0 °C ± 10.7 °C Ta (mean ± SD): Synthetic grass with HydroChill = 35.2 °C ± 1.1 °C RH (mean ± SD): Synthetic grass with HydroChill = 15.0% ± 1.6% Tg (mean ± SD): Synthetic grass with HydroChill = 48.3 °C ± 0.9 °C Vapor pressure (mean ± SD): Synthetic grass with HydroChill = 8.5 mb ± 0.8 mb WBGT (mean ± SD): Synthetic grass with HydroChill: 27.5 °C ± 0.4 °C Wind velocity (mean ± SD): Synthetic grass with HydroChill: 2.8 m^**.**^s^−1^ ± 1.6 m^**.**^s^−1^ Incoming solar radiation (mean ± SD): Synthetic grass with HydroChill = 841 W.m^−2^ ± 50.4 W.m^−2^ Outgoing solar radiation (mean ± SD): Synthetic grass with HydroChill = 58 W.m^−2^ ± 1.2 W.m^−2^ Outgoing long wave solar radiation (mean ± SD): Synthetic grass with HydroChill: 551 W.m^−2^ ± 7.1 W.m^−2^ Albedo: Synthetic grass with HydroChill: 6.9% Surface temperature (mean ± SD): Natural grass = 32.8 °C ± 0.8 °C Ta (mean ± SD): Natural grass = 32.4 °C ± 0.9 °C RH (mean ± SD): Natural grass = 13.1% ± 1.2% Tg (mean ± SD): Natural grass = 45.5 °C ± 1.0 °C Vapor pressure (mean ± SD): Natural grass = 6.4 mb ± 0.6 mb WBGT (mean ± SD): Natural grass = 25.3 °C ± 0.4 °C Wind velocity (mean ± SD): Natural grass = 1.5 m^**.**^s^−1^ ± 1.1 m^**.**^s^−1^ Incoming solar radiation (mean ± SD): Natural grass = 892 W.m^-2^ ± 44.0 W.m^−2^ Outgoing solar radiation (mean ± SD): Natural grass = 113 W.m^−2^ ± 3.0 W.m^−2^ Outgoing longwave solar radiation (mean ± SD): Natural grass = 514 W.m^−2^ ± 3.3 W.m^−2^ Albedo: Natural grass = 12.6% Surface temperature (mean ± SD): Natural grass = 33.3 °C ± 1.24 °C Ta (mean ± SD): Natural grass = 34.9 °C ± 1.2 °C RH (mean ± SD): Natural grass = 14.2% ± 1.1% Tg (mean ± SD): Natural grass = 47.3 °C ± 1.5 °C Vapor pressure (mean ± SD): Natural grass = 19.1 mb ± 0.3 mb WBGT (mean ± SD): Natural grass = 26.9 °C ± 0.3 °C Wind velocity (mean ± SD): Natural grass = 3.7 m^**.**^s^−1^ ± 1.4 m^**.**^s^−1^ Incoming solar radiation (mean ± SD): Natural grass = 956.0 W.m^−2^ ± 26.2 W.m^−2^ Outgoing solar radiation (mean ± SD): Natural grass = 112 W.m^−2^ ± 2.9 W.m^−2^ Outgoing longwave solar radiation (mean ± SD): Natural grass = 508 W.m^−2^ ± 14.0 W.m^−2^ Albedo: Natural grass: 11.7% Surface temperature (mean ± SD): Indoor synthetic grass = 27.0 °C ± 0.2 °C Ta (mean ± SD): Indoor synthetic grass = 27.7 °C ± 0.6 °C RH (mean ± SD): Indoor synthetic grass = 27.6% ± 0.6%
(Xiao and Cao 2013)	Surface temperature (range): Synthetic grass = 38.0–55.5 °C Surface temperature (range): Natural grass 26.4–32.3 °C Synthetic grass surface temperature: 9:20 am: 40.1 °C; 10:20 am: 48.3 °C; 11:20 am: 55.5 °C; 15:00: 48.7 °C; 16:00: 42.2 °C; 17:00: 38.0 °C Natural grass surface temperature: 9:20 am: 29.7 °C; 10:20 am: 28.7 °C; 11:20 am: 32.3 °C; 15:00: 31.5 °C; 1600: 30.9 °C; 1700: 26.4 °C

Statistical significance (*p* < 0.05) was reported underneath the extracted data if studies included such analysis. ***Key***. *Ta* air temperature, *RH* relative humidity, *WBGT* wet-bulb-globe temperature, *Td* dry-bulb temperature, *Tg* globe temperature, and *Tw* wet-bulb-temperature

### Outcome measures

The following definitions describe the included environmental parameters. Air temperature is the temperature of the air 1.2–1.5 m directly above the field. The International Olympic Committee recommends these heights to represent an athlete’s ‘field of play’ (Racinais et al. 2022); therefore, only studies measuring air temperatures within this range are included in the narrative synthesis. Humidity refers to the mass concentration or density of water vapour in the environment; however, it is often expressed as relative humidity, which is the ratio between the partial pressure of water vapour to the saturated water vapour pressure (Parsons 2002). Several measures of radiation are included in this review, including the radiant or mean radiant temperature, which can be defined by the temperature of an enclosure containing a black sphere that would have the same radiation as the surrounding environment, which is calculated by measuring the globe temperature (i.e. a measure of solar radiation; Parsons 2002). Surface temperature refers to the temperature of the ground on the field. The wet-bulb-globe temperature (WBGT) is a heat stress index calculated using natural wet-bulb, air, and globe temperatures (Dehghan et al. 2012). Finally, the heat index is another heat stress parameter representing the temperature a human body ‘feels’ in an environment (Liu and Jim 2021).

## Results

### Study identification

**Fig. 1:**
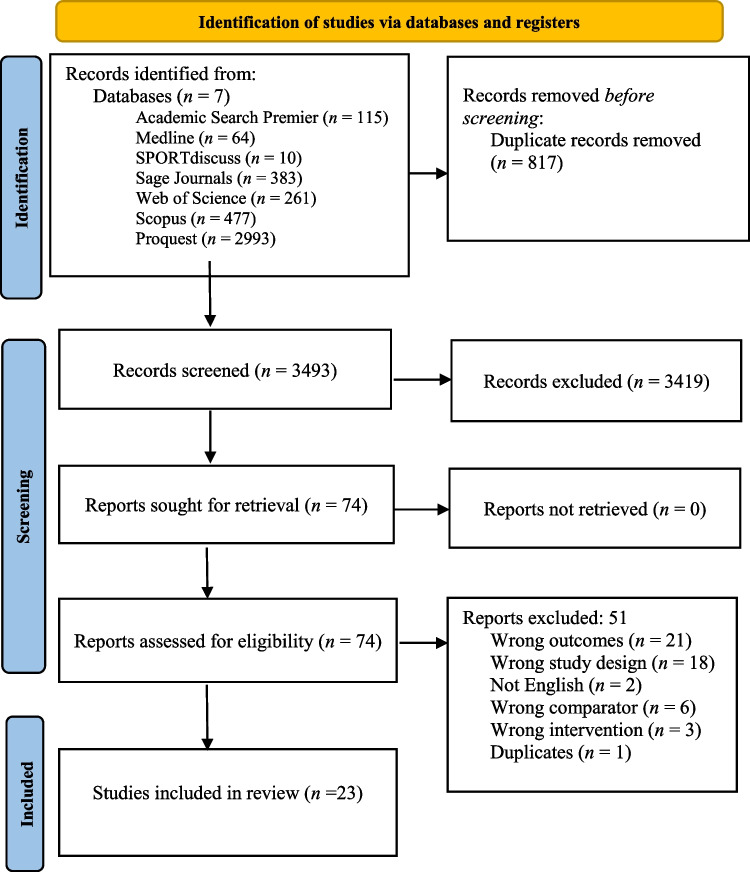
Prisma flow diagram of the screening results

A PRISMA flow chart of the search and screening process is presented in Fig. 1.

### Characteristics of included studies

**Fig. 2:**
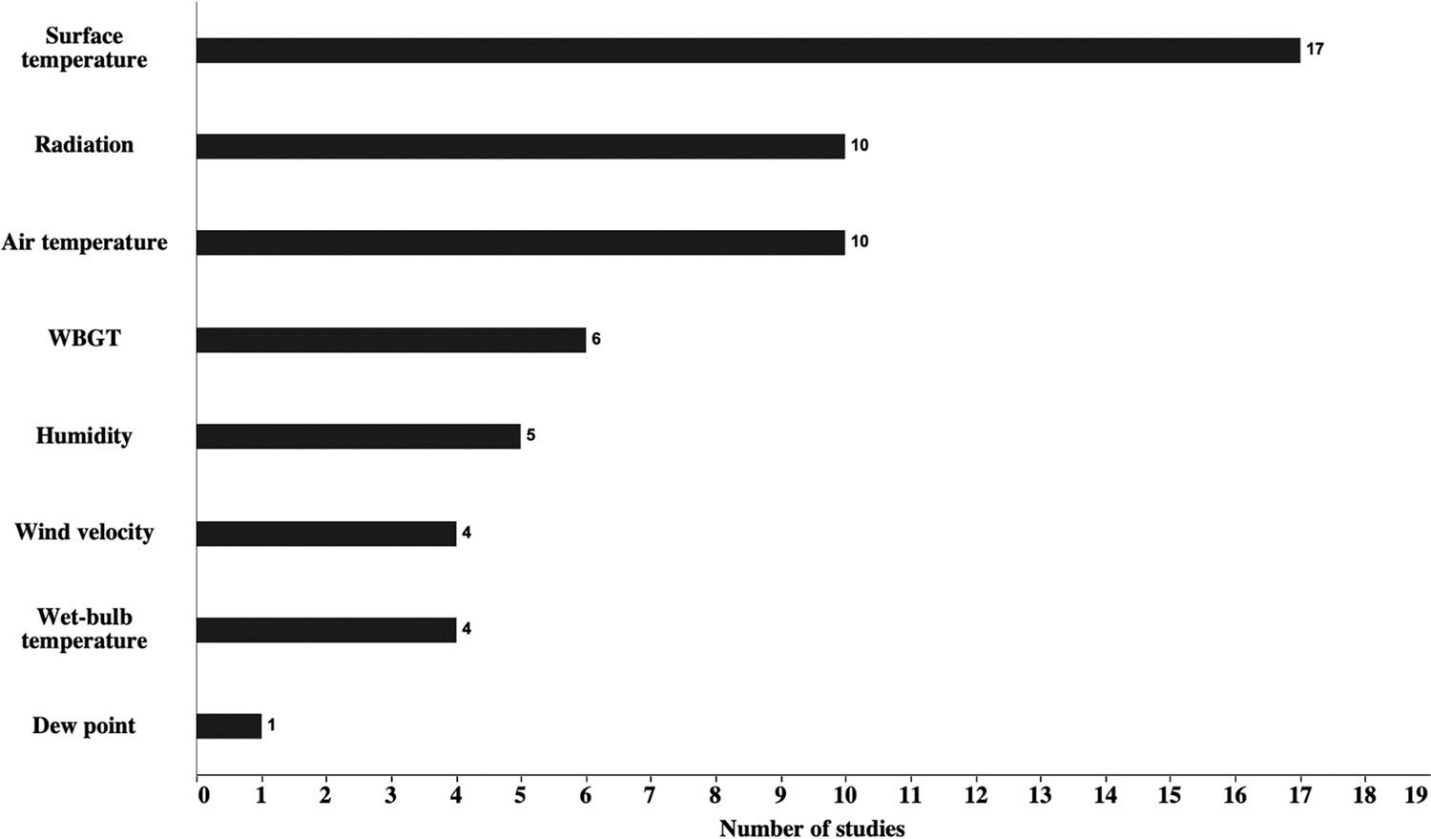
The frequency of the different environmental parameters that were measured across all studies

A range of environmental parameters were investigated. The frequency of the different environmental parameters that were measured across all studies is illustrated in Fig. 2. A summary of the study characteristics, including the surface types, recorded environmental parameters, environmental conditions, recording instruments and the measurement heights of the measured environmental parameters, is presented in Supplementary file 2.

The studies were conducted on full-sized fields (*n* = 16; 70%) or smaller plots (*n* = 7; 30%) of varying sizes (range: 1.4–32 m^2^). Studies were conducted in a range of locations and ambient temperatures, with fifteen (65%) studies performed in ambient temperature conditions > 30 °C, nine (39%) in temperatures between 20 and 20.9 °C, two (9%) between 0 and 19.9 °C, and six (26%) in conditions not reported (Kandelin et al. 1976; Ramsey 1982; Xiao and Cao 2013; Thoms et al. 2014; Pryor et al. 2017; Wardenaar et al. 2022).

### Methodological quality

The results of the methodological quality appraisal are presented in Supplementary file 3. Overall, included studies performed well on the quality appraisal; however, reporting bias, where studies measured a range of parameters, but selectively reported only a subset of those which were measured, was the most common cause of bias.

### Environmental parameters

The results of environmental parameter measurements across all tested surfaces within individual studies are presented in Table 1. Study results are summarised throughout this section via narrative synthesis.

#### Air temperature

Only two studies performed statistical analysis and reported significantly higher air temperatures above synthetic grass than natural grass surfaces at heights from 1.2–1.5 m (Kandelin et al. 1976; Shi and Jim 2022). At 1.5 m, researchers observed a mean difference of 0.5 °C (SD not reported) on a third-generation synthetic compared to natural grass (Shi and Jim 2022), and a greater increase of 1.2 °C (SD not reported) was reported over a Tartan Turf synthetic grass surface in a separate study (Kandelin et al. 1976). Seven further studies compared air temperatures without statistical comparisons, with six demonstrating higher air temperatures over synthetic grass surfaces (Ramsey 1982; Jim 2016, 2017; Hardin and Vanos 2018; Liu and Jim 2021; Guyer et al. 2021; Wardenaar et al. 2022). One study reported a 2.8 °C higher air temperature 1.2 m above a third-generation synthetic grass surface with HydroChill than a natural grass field (mean: 35.2 °C ± 1.1 °C vs. 32.4 °C ± 0.9 °C; Wardenaar et al. 2022). However, this study did not record the air temperatures over both surfaces concurrently, meaning that the environmental conditions were likely different. In a separate study, air temperatures were 0.4–0.9 °C higher, 1.2 m above synthetic than natural grass (Guyer et al. 2021). At 1.5 m, temperatures between third-generation synthetic and natural grass surfaces were also similar (maximum: 36.0 °C vs. 34.8 °C) (Jim 2016) and (maximum: 34.4 °C vs. 34.4 °C; Jim 2017). Only one study reported higher air temperatures above natural grass compared to third-generation synthetic grass, which was measured at 1.6 m (mean: 38.8 °C vs. 37.0 °C, SD not reported); however, temperatures were also taken on separate days where different environmental conditions would be expected (i.e. air velocity and globe temperature), potentially explaining the difference in air temperature (Hardin and Vanos 2018). When the air temperature was measured in different sky conditions at 1.5 m, two studies indicated higher air temperatures between surfaces in sunny, cloudy, and overcast conditions (Jim 2017; Liu and Jim 2021). Overall, these studies demonstrated that air temperature differences between synthetic and natural grass surfaces decreased as the temperature measurement height increased (see Table 1) (Jim 2016, 2017). Nevertheless, these findings indicate that air temperatures above synthetic grass surfaces are 0.5–1.2 °C higher than over natural grass.

#### Radiation

Four studies measured the globe temperature on synthetic and natural grass surfaces (Ramsey 1982; Grundstein and Cooper 2020; Liu and Jim 2021; Wardenaar et al. 2022). One study reported significantly higher globe temperatures 1.2 m above third-generation synthetic compared to natural grass (mean: 48.3 °C ± 0.9 °C vs. 45.5 °C ± 1.0 °C) (Wardenaar et al. 2022) but is should be noted that these were measured on separate days with different environmental conditions that likely influenced the black globe. In a separate study, the median globe temperature was 1.4–1.5 °C higher above third-generation synthetic grass than natural grass (Grundstein and Cooper 2020), but the study did not provide data to verify if the air temperature and wind velocity were similar, which can impact the black globe. Interestingly, another study, which did not perform statistical comparisons between surfaces, reported similar maximum globe temperatures 1.5 m above a third-generation synthetic grass surface compared to a natural grass field for sunny (53.5 °C vs. 53.2 °C), cloudy (48.7 °C vs. 50.8 °C) and overcast (42.9 °C vs. 43.4 °C) sky conditions (Liu and Jim 2021). When the mean radiant temperature was measured in a separate study, researchers reported a significantly higher mean radiant temperature difference 1.5 m above a third-generation synthetic grass surface than a natural grass field in sunny (3.4 °C) and cloudy (0.8 °C) conditions but not in overcast conditions (0.4 °C; Shi and Jim 2022). The differences in mean radiant temperature were in conjunction with higher air temperatures (> 0.5 °C) over the synthetic than the natural grass surface (Shi and Jim 2022).

#### Humidity

Five studies measured the relative humidity above synthetic and natural grass surfaces (Xiao and Cao 2013; Petrass et al. 2014b; Liu and Jim 2021; Guyer et al. 2021; Wardenaar et al. 2022). The raw relative humidity data are provided in Table 1**.** Given the potential confounding factor for differences in air temperature, the relative humidity was converted to absolute humidity for more accurate comparisons. Absolute humidity was 10.21–12.13 g^**.**^m^3^ vs. 9.66–12.05 g^**.**^m^3^ directly above natural and third-generation synthetic grass at two separate locations in one study (Petrass et al. 2014b). In another study, the absolute humidity was similar directly above natural and synthetic grass with HydroChill across in June (mean: 5.39 g^**.**^m^3^ vs. 5.10 g^**.**^m^3^) and August (mean: 10.21 g^**.**^m^3^ vs. 10.25 g^**.**^m^3^; Guyer et al. 2021). In a separate study, the mean relative humidity could not be converted to absolute humidity, and was measured above third-generation synthetic and natural grass surfaces in different sky conditions (Liu and Jim 2021). The largest mean difference was on natural grass, which was higher in sunny (3.9%), cloudy (2.2%) and overcast (3.6%) conditions (SD not reported; Liu and Jim 2021). However, there was an exception, with the mean humidity higher above third-generation synthetic than natural grass surfaces in a separate study (outdoor synthetic: 5.99 g^**.**^m^3^ vs. outdoor natural: 4.52 g^**.**^m^3^) and (indoor synthetic: 7.39 g^**.**^m^3^ vs. outdoor natural: 5.59 g^**.**^m^3^), but this study did not measure the humidity on separate days (Wardenaar et al. 2022). These findings suggest that the humidity is similar above synthetic and natural grass surfaces.

#### Wind velocity

While four studies recorded the wind velocity above synthetic and natural grass surfaces (Petrass et al. 2014b; Guyer et al. 2021; Wardenaar et al. 2022; Shi and Jim 2022), only two used statistical analyses to compare results between surfaces (Wardenaar et al. 2022; Shi and Jim 2022). The wind velocity varied across these studies, with only one investigation reporting a significantly higher wind velocity over synthetic grass with HydroChill compared to natural grass (mean: 2.8 m^**.**^s^−1^ ± 1.57 m^**.**^s^−1^ vs. 1.5 m^**.**^s^−1^ ± 1.1 m^**.**^s^−1^); however, the wind velocity was measured on separate days (Wardenaar et al. 2022). Whereas in a separate study, surface types had no significant differences (mean: 0.42 m^**.**^s^−1^; Shi and Jim 2022). In studies where no statistical comparisons were performed, researchers reported similar wind velocities above synthetic and natural grass surfaces (location 1 with Coolclimate synthetic turf mean: 7.65 km^**.**^h^−1^ ± 6.49 km^**.**^h^−1^ vs. 8.10 ± 6.39 km^**.**^h^−1^; location 2 with third-generation synthetic grass mean: 8.28 km^**.**^h^−1^ ± 5.49 km^**.**^h^−1^ vs, 7.43 km^**.**^h^−1^ ± 4.19 km^**.**^h^−1^; Petrass et al. 2014b) and (mean in June: 1.2 m^**.**^s^−1^ ± 0.81 m^**.**^s^−1^ vs. 1.3 m/s^−1^ ± 0.90 m^**.**^s^−1^; August: 1.1 ± 0.83 vs. 1.3 m^**.**^s^−1^ ± 0.90 m^**.**^s^−1^; Guyer et al. 2021).

#### Unified heat stress indices

Six studies compared the WBGT on synthetic and natural grass surfaces, demonstrating similar WBGT on both surface types (Ramsey 1982; Pryor et al. 2017; Grundstein and Cooper 2020; Liu and Jim 2021; Guyer et al. 2021; Wardenaar et al. 2022). It should be noted that although these studies measured the WBGT, only two studies accurately recorded the air, natural wet bulb and black globe temperature (Ramsey 1982; Liu and Jim 2021), which are required to calculate the WBGT. The other studies used handheld heat stress monitors that estimate the natural-wet bulb temperature based on the relative humidity (Havenith and Fiala 2016), which is then used to calculate the WBGT (Pryor et al. 2017; Grundstein and Cooper 2020; Guyer et al. 2021; Wardenaar et al. 2022). When the WBGT was accurately measured, the mean WBGT was very similar between third-generation synthetic and natural grass in sunny (mean: 31.27 °C vs. 31.42 °C; SD not reported), cloudy (mean: 29.44 °C vs. 29.47 °C; SD not reported) and overcast (mean: 27.84 °C vs. 27.77 °C; SD not reported) conditions (Liu and Jim 2021). Similarly, researchers reported a mean WBGT difference of 0.52 ± 0.8 °C between a Tartan Turf and natural grass surface (Ramsey 1982). This was consistent across studies that used handheld heat stress monitors, with one study reporting no significant mean differences between a third-generation synthetic grass surface (0.09 ± 2.33 °C) or Astroturf (0.61 ± 1.88 °C; Pryor et al. 2017). Similarly, another study demonstrated no significant differences in the mean WBGT between synthetic grass with HydroChill and natural grass (30.1 °C vs. 29.6 °C, SD not reported; Guyer et al. 2021). In another study, there were no significant differences between the median WBGT of a third-generation synthetic and natural grass surface in the morning, midday or afternoon (Grundstein and Cooper 2020). Finally, a separate measure of heat index was higher on a third-generation synthetic grass surface than natural grass on sunny (7.80 °C), cloudy (2.53 °C) and overcast (6.38 °C) days (SD not reported) (Liu and Jim 2021); however, no statistical comparisons were performed. As such, these data demonstrate that the calculated heat stress parameters may not be different between synthetic and natural grass surfaces.

#### Surface temperature

Thirteen studies compared surface temperatures between synthetic and natural grass surfaces, including five that reported significantly higher surface temperatures on synthetic than natural grass in hot environmental conditions (Kandelin et al. 1976; Petrass et al. 2014b; Twomey et al. 2016; Guyer et al. 2021; Shi and Jim 2022). This was consistent across a first-generation synthetic grass vs. natural grass surface (mean daily maximum: 49.3 °C vs. 39.9 °C, SD not reported; Kandelin et al. 1976) and when third-generation synthetic grass surfaces were compared with natural grass fields in another study (mean: 46.3 °C ± 15.6 °C vs. 35.1 °C ± 10.0 °C) (Twomey et al. 2016) and in a separate study (mean: 46.0 °C ± 14.4 °C vs. 23.8 °C ± 5.3 °C; Petrass et al. 2014b). Three other studies also observed higher surface temperatures on third-generation synthetic grass surfaces than natural grass, with maximum temperature differences on different days of 20.9–36.5 °C (Jim 2017) and 32.1–37.6 °C (Jim 2016). Surface temperatures on third-generation synthetic grass with Cool climate (mean: 40.1 °C ± 12.8 °C vs. 27.6 °C ± 7.4 °C; Petrass et al. 2014b) turf fibres or HydroChill (mean: 67.0 °C ± 10.7 °C vs. 33.3 °C ± 1.1 °C; Wardenaar et al. 2022) were still significantly higher than natural grass. In two of the studies, synthetic and natural grass maximum surface temperatures were compared in clear, cloudy, and overcast conditions. The maximum surface temperature on synthetic grass surfaces exceeded natural grass by 25.8 °C (Liu and Jim 2021) and 36.5 °C (Jim 2017) in clear conditions and by 21.9 °C (Liu and Jim 2021) and 29.7 °C (Jim 2017) in cloudy conditions. The smallest differences in maximum surface temperatures were in overcast conditions, with synthetic grass temperatures still exceeding natural grass by 20.9 °C (Liu and Jim 2021) and 13.7 °C (Jim 2017). Overall, surface temperatures were consistently higher on synthetic surfaces than natural grass across all studies, regardless of the environmental conditions, turf fibres used or the sky conditions.

Four studies performed comparisons between surface temperatures on different types of synthetic surfaces. Full-sized synthetic surfaces containing SBR infill had significantly higher surface temperatures than fields with TPE infill (mean: 61.2 °C ± 6.5 °C vs. 58.0 ± 5.0 °C; Villacañas et al. 2017). Similarly, synthetic grass plots with SBR infill were significantly hotter than plots with TPE infill (mean: 53.5 °C vs. 45.6 °C, SD not reported; Petrass et al. 2014a). In the same study, the influence of shock pads on synthetic grass plot surface temperatures were investigated, and significantly higher surface temperatures were reported on a synthetic plot with a shock pad compared to without (mean: 50.3 °C vs. 48.5 °C, SD not reported) (Petrass et al. 2014a). A separate study demonstrated no difference in surface temperature on synthetic plots with different infill ratios of SBR and sand (Thoms et al. 2014), and another observed a similar surface temperature on a synthetic grass plot coloured red compared to green (mean: 58.2 °C ± 14.5 °C vs. 56.6° ± 14.3 °C; (Pfautsch et al. 2022), but no statistical comparisons were performed. Accordingly, synthetic grass surfaces with TPE infill or without a shock pad were cooler than surfaces with SBR rubber infill, however, it is unclear whether the different features also affect other environmental parameters essential for human heat exchange.

## Discussion

This systematic review aimed to (i) determine if there are differences in the thermal environment, and (ii) determine if there are differences in the thermal environment between different types of synthetic grass surfaces. Overall, the data demonstrated that measures of air temperature and surface temperature were significantly higher on synthetic grass surfaces than natural grass. Synthetic grass surfaces with TPE infill, Cool climate turf fibres, HydroChill product and without a shock pad, are cooler than surfaces with SBR infill or a shock pad, but it is unclear what influence these features have on the thermal environment. Based on the published research, more data is needed to produce a meta-analysis on these comparisons and to determine if different synthetic grass surfaces interact differently with the thermal environment.

It is important to note that this review included comparisons of air temperature, radiant temperature, humidity, and wind velocity because these are the four key environmental parameters that directly influence human heat exchange pathways and the subsequent risk of heat stress and hyperthermia for a person exercising/playing sport on the different surfaces (Cramer and Jay 2016; Chalmers et al. 2020). However, among the 23 included studies, only two studies measured all four of these parameters (Hardin and Vanos 2018; Liu and Jim 2021). Instead, most of the studies focussed on surface temperature, which was consistently higher on synthetic than natural grass. Synthetic grass surfaces have a lower albedo (i.e. the ratio of short-wave radiation reflected by a surface to the total incoming solar radiation; Nazarian et al. 2017) than natural grass (Jim 2016, 2017; Loveday et al. 2019; Liu and Jim 2021; Carvalho et al. 2021; Wardenaar et al. 2022). The low albedo coincides with a lower specific heat capacity of the turf fibres and the infill material, meaning that it requires less energy to heat the surface (Jim 2016, 2017). Natural grass has a high specific heat capacity due to natural turf consisting of approximately 70% moisture by weight (Devitt et al. 2007), promoting cooling through evapotranspiration (Jim 2016, 2017; Liu and Jim 2021). Accordingly, these differences can explain the different surface temperatures reported between synthetic and natural grass surfaces.

Researchers have raised concerns regarding an increased risk of hyperthermia and heat illness from high synthetic grass surface temperatures. These concerns are based on the premise that heat could transfer from the hot surface into the body via foot conduction (Buskirk et al. 1971). However, conduction is considered negligible in outdoor environments unless there is direct skin contact with the surface for prolonged durations (i.e. standing stationary without shoes or lying on the surface; Cramer and Jay 2016, 2019). Individuals who are exercising/playing sport are constantly moving, meaning that the foot contact time is likely insufficient for meaningful heat gain to occur via conduction, especially considering that they are wearing shoes that act as an insulator (Cramer and Jay 2016, 2019). Instead, it is likely the amount of human absorbed radiation from the surface that could increase the level of heat stress. Indeed, studies have demonstrated that synthetic grass surfaces emit more terrestrial radiation than natural grass (Jim 2016, 2017), and as such, athletes may be exposed to increased radiation, which highlights the importance of the mean radiant temperature as an outcome measure in the current literature and in future research. Due to the focus on surface temperature and the lack of research on mean radiant temperature, it is difficult to conclude whether synthetic grass sports surfaces can alter the thermal environment in a way that has a meaningful impact on the four environmental parameters essential for human heat exchange. Accordingly, the four key environmental parameters of air temperature, mean radiant temperature, humidity, and wind velocity should be the focus of future investigations on this topic.

Air temperatures were reported to increase by 0.5–1.2 °C on synthetic grass compared to natural grass. The higher air temperatures over synthetic compared to natural grass can likely be explained by synthetic grass having a lower albedo leading to increased energy storage on the surface, which is then transformed into heat and then moved into the air above the surface via longwave radiation (Jim 2016). This is further supported by several studies reporting that synthetic grass surfaces emitted higher levels of longwave radiation than natural grass (Jim 2016, 2017; Hardin and Vanos 2018; Liu and Jim 2021; Carvalho et al. 2021; Wardenaar et al. 2022), likely contributing to the slightly higher air and mean radiant temperatures above synthetic compared to natural grass surfaces. This difference in air temperature raises an interesting question; can a 0.5–1.2 °C higher air temperature above synthetic grass can cause a meaningful change in heat stress for a person exercising/playing sport on that surface? To put this into perspective, the Federation Internationale de Football Association has an exertional heat-illness risk evaluation based on the air temperature (Mountjoy et al. 2012). This evaluation has three risk categories (i.e. moderate, high and extreme), and the air temperature range within each risk category is ~ 6.0 °C (Mountjoy et al. 2012). As such, a 1.2 °C difference in air temperature above a synthetic grass surface is one-fifth of a change in a category; therefore, in this context, we do not believe that this difference would lead to a meaningful increase in risk. However, it should be noted that this is an empirical model and given that heat stress involves a complex interplay between physiological and environmental parameters, this example should be interpreted cautiously because individuals could respond differently to these temperature changes based on many variables, for example, their fitness level and clothing worn.

This review has demonstrated no differences in the WBGT between surface types, which is one of the calculated indices providing an overall assessment for the potential for differences in heat stress for a person. The WBGT is calculated using the globe temperature, air temperature and natural wet-bulb temperature. Given that studies measuring WBGT reported differences in the environmental parameters required to calculate the WBGT, it is likely the differences were either not large enough to change the WBGT, or in some instances, different parameters were higher on different surfaces, which neutralised the overall result. This was observed in one study, where the globe and air temperatures were higher over synthetic than natural grass in some instances, while the dew point was higher over the natural than synthetic grass, resulting in similar WBGT between surfaces (Grundstein and Cooper 2020). Further, only two studies accurately calculated the WBGT by directly measuring the wet-bulb temperature, while the others estimated it by measuring the relative humidity, which was then used to calculate the WBGT. Unfortunately, any device calculating the WBGT that does not directly measure each component of the index is limited (Chalmers et al. 2020) and therefore, further research is required.

This review also demonstrated a similar humidity and air velocity over synthetic and natural grass surfaces across a range of locations and climates. As natural grass often has more moisture content than synthetic grass surfaces (Devitt et al. 2007; Liu and Jim 2021), there is an increased transfer of moisture to the air via evapotranspiration, which can elevate the humidity surrounding the surface (Jim 2016; Grundstein and Cooper 2020). However, differences in humidity between synthetic and natural grass surfaces were minimal. These small differences are likely due to the atmosphere being an efficient regulator of environmental parameters, meaning that large differences in humidity are effectively minimised quickly (Brown and Gillespie 1997). Similar air velocity over synthetic and natural grass was expected, given that there is no proposed mechanism whereby the surface could impact the air velocity. The only differences reported were due to suboptimal study design in some studies, where the air velocity was measured on different days between surfaces.

There were several methodological limitations related to the measurement techniques and data presentation across the included studies. One major limitation was the small sample sizes (i.e., the number of unique surfaces and the number of days of data collection on each). Studies often only recorded environmental parameters from two fields (see Table 1) and with limited reporting of the local temperature and sky conditions, making it difficult to generalise the results. Some studies increased their sample size by testing the surfaces in the form of smaller-sized plots, however, it is unclear if small plots mimic the microclimate of a full-sized field. It can be assumed that a larger field will have a greater impact on the thermal environment than a small plot, as greater air volume with higher temperature and humidity would be diluted at a slower rate. Another issue identified was that the measurement height above the surface at which the environmental parameters were measured varied across the included studies. While the narrative synthesis only discussed studies that used a height recommended by the International Olympic Committee (approximately 1.2–1.5 m; Racinais et al. 2022), measurements within several studies were taken between 0.15 to 1.6 m above the surface. Unfortunately, certain environmental parameters (e.g., air temperature) can fluctuate depending on the measurement height (see Table 1), which makes it difficult to pool the data and draw meaningful conclusions.

The sampling frequency used and the reporting of data were also not consistent across the included studies. Researchers collected the environmental parameters between 15-minute to 1-hour intervals and reported these data as a median, mean or minimum and maximum value. The variability with the sampling intervals and data reporting makes it difficult to contextualise the results, for example, when a study reported a maximum surface temperature collected at 1-hour intervals, it is unknown how long the temperature was present. Therefore, it cannot be determined if the surface temperature could cause a meaningful change to other environmental parameters. The critical appraisal also flagged that some studies may have selectively reported their data, with several investigations presenting a minimum and maximum surface temperature despite presenting the mean for other variables, which suggests that the data was presented in this way to inflate the differences between surfaces. It should be noted that the results of these studies have the potential to influence policymaking, and it is imperative that authors are transparent about their methods and reporting.

A secondary aim of this review was to determine if there are differences in the thermal environment between different types of synthetic grass surfaces. Most of the included studies assessed only modern-generation synthetic grass surfaces, with only three studies incorporating (what we believed to be) first-generation synthetic surfaces. This made it difficult to compare if there are differences in the thermal environment between different synthetic grass surface generations, particularly given that most of these studies measured components of the thermal environment at different heights or using different equipment. Considering that first-generation synthetic surfaces are now redundant, it is perhaps more important to compare modern-generation synthetic surfaces with different features, for example, the use of different infill materials, shock pads or fibre types. One study demonstrated that third-generation synthetic surfaces with SBR infill or a shock pad had higher surface temperatures than those without these features, or with TPE infill (Petrass et al. 2014a). These differences can be explained by SBR infill and the rubber shock pads having a greater heat absorption capacity than TPE infill (Petrass et al. 2014a; Villacañas et al. 2017). Other studies demonstrated that third-generation surfaces with Cool climate turf fibres or surfaces containing HydroChill had lower surface temperatures than traditional third-generation synthetic grass surfaces (Petrass et al. 2014b; Wardenaar et al. 2022). Cool climate turf fibres are designed to reflect sunlight and disperse heat into the surrounding environment, thus reducing surface temperatures (Petrass et al. 2014b). However, the increased reflectance could elevate the mean radiant temperature directly above the surface, potentially increasing the capacity for heat gain for exercising individuals. When the HydroChill product is added to a synthetic surface it promotes moisture storage within the infill, attenuating the rise in surface heat (Wardenaar et al. 2022). Notably, this increased moisture may elevate the humidity around the surface, hindering the sweat evaporation capacity for exercising individuals. Hence, these studies were focused on the influence of these features on the surface temperature rather than the key parameters that influence heat exchange, and therefore, the effect of these features on other measures of the thermal environment is unclear.

## Future research directions

While studies have measured various environmental parameters over synthetic grass surfaces since the 1970’s (Kandelin et al. 1976; Ramsey 1982), only two studies have provided data for the environmental parameters essential for human heat exchange over synthetic and natural grass surfaces (Hardin and Vanos 2018; Liu and Jim 2021). Most of the research has focused on surface and air temperatures, which are important but these parameters alone do not holistically determine the impact synthetic grass has on the thermal environment. Given that individuals exercising on synthetic grass surfaces are exposed to more longwave radiation than on natural grass (Jim 2016, 2017; Liu and Jim 2021), the influence these surfaces have on the mean radiant temperature should be further investigated and then combined with the environmental parameters essential for human heat exchange to assess the level of heat stress. Without this holistic view of the thermal environment, it is difficult to determine if synthetic grass surfaces can alter the thermal environment and increase the risk of human heat stress. Future research is needed to measure the air temperature, mean radiant temperature, humidity, and wind velocity over various synthetic grass surfaces across a range of environmental conditions. These studies should present the time course of the data at short intervals throughout the measurement period and determine if certain times of day present a greater risk. Importantly, these environmental parameters could be used to predict the level of heat stress using human modelling (i.e., partitional calorimetry).

Further, human data is needed to determine the effects of synthetic surfaces on human heat stress. Only one study has demonstrated that synthetic grass can increase markers of heat stress (e.g. skin temperature) compared to natural grass, but there was no observed effect on core body temperature (Wardenaar et al. 2022). However, further research is required to confirm these findings in well-controlled experiments and in different populations. Children and youth are likely to be at increased risk due to their stature being closer to the surface, where the air is the hottest (Jim 2016, 2017; Liu and Jim 2021).

It has been previously suggested that synthetic grass surfaces can contribute to the heat island effect, which occurs when areas experience higher temperatures than their surrounding environment (Claudio 2008). Since synthetic grass surfaces are rapidly increasing, particularly in residential areas (i.e. lawns and parks), this could have implications on urban human thermal comfort, which refers to an individual’s satisfaction with the thermal environment and allows the quantification of how an average person ‘feels’ according to the environmental conditions they are exposed to (Zhao et al. 2021). Studies have reported that rapid urbanisation can decrease thermal comfort (Ren et al. 2022a) and cause physiological changes (Ren et al. 2022b). This is a concern because athletes can change their behaviour to avoid excessive fatigue in the heat if they perceive the environmental conditions as threatening, which can negatively affect performance (Périard et al. 2014; Nassis et al. 2015). Since this review demonstrates that there may be differences in some of the environmental parameters between synthetic and natural grass, future research should also assess whether these changes are enough to influence human thermal comfort.

Studies have investigated methods to reduce surface temperatures on synthetic grass sports surfaces. Indeed, this systematic review suggests that studies should not exclusively focus on surface temperatures when discussing the human heat stress risk to an athlete playing sport on a synthetic grass surface, therefore, further practical recommendations are not appropriate until further research has been performed, particularly investigating the effect on human absorbed radiation. Nevertheless, it can be assumed that the largest changes to the environmental parameters that govern human heat exchange occur when surface temperatures increase. As such, further research is required on modern synthetic grass surfaces, which have benefited from technology that can reduce surface temperatures in warm conditions (Petrass et al. 2014b). Synthetic grass manufacturers have introduced different infill materials (e.g., SBR, TPE and cork), and the use of TPE infill results in significantly cooler surface temperatures than SBR infill (Petrass et al. 2014a; Villacañas et al. 2017). Therefore, the use of TPE infill is recommended to reduce surface heat, however, other infill options including, cork and different coloured rubber have not been investigated extensively and future research should determine if these influence the surface temperature and the thermal environment.

As explained in the Surface temperature section two studies have demonstrated significantly lower surface temperatures on synthetic grass surfaces with Cool climate and HydroChill technology than traditional third-generation synthetic and natural grass surfaces (Petrass et al. 2014b; Wardenaar et al. 2022). However, it is unclear if the increased reflectance from Cool climate turf fibres or increased stored moisture from HydroChill can affect the thermal environment above the surface and potentially increase the risk of greater heat transfer to a person on the surface. Therefore, future research should investigate whether these modifications result in differences in the four key environmental parameters of human heat exchange, as well as differences in markers of heat stress in exercising humans before these new surfaces are recommended.

While sports facilities with synthetic grass surfaces often use water irrigation to reduce surface temperatures (McNitt et al. 2008; Fleming 2011; Kanaan et al. 2020), the influence this has on the surrounding environmental parameters is unknown. Adding water to a surface may elevate the humidity surrounding the surface, which is concerning since athletes competing in humid environmental conditions have impaired evaporative cooling capacity (Cramer and Jay 2016; Périard et al. 2021), which increases the risk of heat stress and hyperthermia. Therefore, future research is required to perform a comprehensive assessment of the effect of water irrigation on synthetic surfaces to reduce surface temperatures, including the impacts on all important environmental parameters and the effects on exercising humans in warm conditions.

## Conclusion

After collating the environmental parameters measured on synthetic grass surfaces, it is evident that studies have focused on surface temperatures, and there is limited published research on other, more important environmental parameters. Only two studies have comprehensively measured and provided data for the environmental parameters essential for human heat exchange, and as such, it is difficult to conclude if synthetic grass surfaces can alter the thermal environment compared to natural grass, especially considering the wide range of synthetic surfaces in existence, and across the whole range of possible environmental conditions on any day, in any location. Nevertheless, the included studies suggest that both the air temperature and surface temperature can be higher on synthetic grass than natural grass, but it is unknown if these differences are a concern to human heat stress. The review also observed that different synthetic grass turf infills (e.g. TPE or HydroChill) and fibres (e.g. Cool climate) can reduce surface temperatures, but it is unknown what influence these different synthetic grass features have on the thermal environment as a whole. Accordingly, this systematic review demonstrates that further investigations with improved and consistent measurement techniques and data reporting methods are required to determine if synthetic grass surfaces can meaningfully affect the thermal environment and the capacity for human heat.

### Supplementary information

ESM 1 (DOCX 20 kb)

ESM 2 (DOCX 22 kb)

ESM 3 (DOCX 18 kb)

## Data Availability

The datasets generated and/or analysed during the current study are available in Supplementary File 1, 2 and 3.
