# Supplementary material for: The effect of synthetic grass sports surfaces on the thermal environment: A systematic review
Source: Int J Biometeorol. 2024 May 1;68(7):1235–52. doi: 10.1007/s00484-024-02679-5 (PMC11272752; doi:10.1007/s00484-024-02679-5)
Supplement: Supplementary file 1 — (DOCX 20 kb) [file 484_2024_2679_MOESM1_ESM.docx]

## Article title: The Effect of Synthetic Grass Sports Surfaces on the Thermal Environment: A Systematic Review

**Journal name:** International Journal of Biometeorology
**Author names:** Gurpreet Singh^1^, Benjamin Peterson^2^, Ollie Jay^3^, Christopher. J. Stevens^1^

**Affiliations:** ^1^Physical Activity, Sport, and Exercise Research Theme, Faculty of Health, Southern Cross University, Coffs Harbour, NSW, Australia

^2^School of Health, Medical and Applied Sciences, Central Queensland University, Australia

^3^Heat and Health Research Incubator, Faculty of Medicine and Health, University of Sydney, Australia

**Email:** Gurpreetsinghphd1@gmail.com

**Supplementary File 1.** Search returns that were excluded after full-text screening and the reason for exclusion

| **Authors** | **Title of search return** | **Reason for exclusion** |
| --- | --- | --- |
| Olsen et al., 2019 | Shade provision in public playgrounds for thermal safety and sun protection: A case study across 100 play spaces in the United States | Wrong outcomes |
| Fleming & Forrester, 2011 | Guest Editorial | Wrong study design |
| Setterbo et al., 2012 | Validation of a Laboratory Method for Evaluating Dynamic Properties of Reconstructed Equine Racetrack Surfaces | Wrong outcomes |
| Golden, 2021 | The contribution of artificial turf to global warming | Wrong study design |
| Kuang, 2020 | Seasonal variation in air temperature and relative humidity on building areas and in green spaces in Beijing, China | Wrong comparator |
| Calderon-Pellegrino et al., 2020 | Influence of artificial turf temperature on physical performance and muscle contractile properties in football players after a repeated-sprint ability test | Wrong outcomes |
| Herdt et al., 2018 | Outdoor thermal comfort during anomalous heat at the 2015 pan American games in Toronto, Canada | Wrong outcomes |
| Claudio, 2008 | Synthetic turf debate takes root | Wrong study design |
| Peterson et al., 2010 | Effect of temperature on race times on a synthetic surface | Wrong intervention |
| Fleming, 2011 | Artificial turf systems for sport surfaces: Current knowledge and research needs | Wrong study design |
| Grundstein et al., 2022 | Are local weather stations a feasible substitute for on-site measurements for heat stress assessment in sports? | Wrong study design |
| Garai et al., 2011 | Air and surface temperature coupling in the convective atmospheric boundary layer | Wrong comparator |
| Lioy et al., 2008 | Artificial turf: Safe or out on ball fields around the world | Wrong study design |
| Reider, 2012 | Gridiron greenery | Wrong study design |
| Yaghoobian et al., 2010 | Modelling the thermal effects of artificial turf on the urban environment | Wrong study design |
| Dorsey et al., 2015 | Mutagenic potential of artificial athletic field crumb rubber at increased temperatures | Wrong outcomes |
| Gorham, 1997 | Actual field performance of synthetic turf as measured over a thirty-year period | Wrong study design |
| BektasBalcik & Filiz, 2014 | Determining the impact of urban components on land surface temperature of Istanbul by using remote sensing indices | Wrong outcomes |
| Forrester, 2014 | Spatial and temporal analysis of surface hardness across a third-generation artificial turf pitch over a year | Wrong outcome |
| Lawrence et al., 2016 | Influence of extrinsic risk factors on national football league injury rates | Wrong outcomes |
| Shooshtarian & Rajagopalan, 2018 | Daytime thermal performance of different urban surfaces: A case study in educational institution precinct of Melbourne | Wrong intervention |
| Aldahir et al., 2014 | A review of sports turf research techniques related to playability and safety standards | Wring study design |
| Zhang et al., 2019 | The effect of xenon lamps radiation on artificial football turf | Wrong outcomes |
| Antoniadis et al., 2018 | Simulation of schoolyard’s microclimate and human thermal comfort under Mediterranean climate conditions: effects of trees and green structures | Wring comparator |
| Stanitski et al., 1974 | Synthetic turf and grass: A comparative study | Wrong outcomes |
| Haider et al., 2018 | Does the environment influence the frequency of concussion incidence in professional football? | Wrong outcomes |
| Strąk et al., 2021 | Safety Comes First: novel styrene butadiene rubber (sbr) and ethylene propylene diene monomer (epdm) surfaces as a response to sport injuries | Wrong outcomes |
| Yoshida et al., 1988 | Spectral reflectance of environmental surfaces for solar radiation | Not English |
| Kanaan et al., 2020 | Water requirements for cooling artificial turf | Wrong comparator |
| Vladamir et al., 2014 | A gis analysis of artificial surfaces impact on urban heat islands of Novi Sad, Serbia | Wrong study design |
| Lulli et al., 2010 | An innovative hybrid natural-artificial sports turf construction system: Plant establishment and playing characteristics | Wrong outcomes & duplicate (n=2) |
| Schmitz, 1988 | Polyurethane-based synthetic surfaces for sports facilities | Wrong study design |
| Jacobsen, 2008 | JCE resources for chemistry and sports | Wrong outcomes |
| Garratt et al., 1973 | Momentum, heat and water vapour transfer to and from natural and artificial surfaces | Wrong outcomes |
| Yoshida et al., 1989 | Solar radiation spectral reflectance of environmental surfaces | Not English & duplicate |
| Gorham et al., 1994 | Actual field performance of synthetic turf as measured over a thirty-year period | Wrong study design & duplicate |
| Schoukens, 2009 | Developments in textile sports surfaces | Wrong outcomes |
| Sánchez-Sánchez et al., 2018 | Effect of structural components, mechanical wear and environmental conditions on the player–surface interaction on artificial turf football pitches | Wrong outcomes |
| Twomey et al., 2016 | Selection and management of sports grounds: Does surface heat matter? | Duplicate |
| Gallardo et al., 2018 | Effect of extrinsic factors and structural components on sport functionality of artificial turf surfaces | Wrong outcomes |
| Twomey et al., 2011 | Challenges in the development of standards for synthetic turf for Australian football and cricket | Wrong outcomes |
| Cheng et al., 2020 | A comprehensive model for estimating heat vulnerability of young athletes | Wrong comparator |
| Tripp et al., 2020 | Comparison of wet bulb globe temperature measured on-site vs estimated and the impact on activity modification in high school football | Wrong outcomes |
| Twomey et al., 2014 | Heat experienced on synthetic turf surfaces: An inevitable or preventable risk? | Wrong study design |
| Breland, 1990 | Performance standards for artificial turf surfaces | Wrong study design |
| Schneider et al., 2014 | Synthetic turf vs. natural grass | Wrong study design |
| WSA, 2004 | Olympic green | Wrong study design |
| Addas et al., 2020 | Utilizing remotely sensed observations to estimate the urban heat Island effect at a local scale: Case study of a university campus | Wrong intervention |
