# Supplementary material for: The effect of synthetic grass sports surfaces on the thermal environment: A systematic review
Source: Int J Biometeorol. 2024 May 1;68(7):1235–52. doi: 10.1007/s00484-024-02679-5 (PMC11272752; doi:10.1007/s00484-024-02679-5)
Supplement: Supplementary file 2 — (DOCX 22 kb) [file 484_2024_2679_MOESM2_ESM.docx]

## Article title: The Effect of Synthetic Grass Sports Surfaces on the Thermal Environment: A Systematic Review

**Journal name:** International Journal of Biometeorology
**Author names:** Gurpreet Singh^1^, Benjamin Peterson^2^, Ollie Jay^3^, Christopher. J. Stevens^1^

**Affiliations:** ^1^Physical Activity, Sport, and Exercise Research Theme, Faculty of Health, Southern Cross University, Coffs Harbour, NSW, Australia

^2^School of Health, Medical and Applied Sciences, Central Queensland University, Australia

^3^Heat and Health Research Incubator, Faculty of Medicine and Health, University of Sydney, Australia

**Email:** Gurpreetsinghphd1@gmail.com

**Supplementary File 2:** *The characteristics of the included studies*

| **Study (year)** | **Surface Types** | **Environmental conditions** | **Environmental parameters** | **Recording instrument(s)**  **(Measurement height)** |
| --- | --- | --- | --- | --- |
| (Bozdogan Sert et al. 2021) | 1 x Synthetic grass field  1 x Natural grass field | Ta mean: 27.8-29.1°C  RH mean: 60.3-71.5%  Wind velocity mean: 1.4-2.1 m**^.^**s^-1^ | Surface temperature | Bosch PTD 1 laser thermometer  (1.5 m) |
| (Carvalho et al. 2021) | 1 x Synthetic grass plot  1 x Natural grass plot: Augustine grass | Ta mean: 22.6-30.6°C  Total solar radiation mean: 12.7-215 MJ.m^-2.^  Wind velocity mean: 1.3-3.1 m**^.^**s^-1^  Water vapour pressure mean: 1.2-2.3 kPa | Surface temperature  Solar radiation | Apogee SS-110 & SS-120  Sonic anemometer Atmos22  Rotronic HC2S3 temperature and humidity probe  (Surface temperature: 0.5 m)  (Environmental parameters: 2 m) |
| (Pfautsch et al. 2022) | 4 x Synthetic grass plots  1 x Natural grass plot | Ta mean: 17-32°C  Solar radiation mean: 0-940 W/m^-2^  Wind velocity mean: <3 m**^.^**s^-1^  RH mean: 67-83%  WBGT mean: 24-29°C | Surface temperature | Teledyne T540 FLIR radiometric infrared camera  (1 m) |
| (Grundstein and Cooper 2020) | 1 x Third-generation synthetic grass field (FieldTurf)  1 x Natural grass field | Ta max: 30.6-32.6°C  Ta min: 17.7-20.2°C. | WBGT  Solar radiation  Dew point | Krestel 5400 heat stress meter  (1.2 m) |
| (Guyer et al. 2021) | 1 x Synthetic grass field, coconut husk, sand and zeolite infill, with HydroChill fibres  1 x natural grass field | Ta mean: 29-36°C  RH mean: 12-30% | Surface temperature  Ambient temperature  Relative humidity  Wind velocity  WBGT | Elekcity Lasergrip 774 IR thermometer  Krestel 5400 heat stress meter  (1.2 m) |
| (Hardin and Vanos 2018) | 1 x Third-generation synthetic grass field  2 x Natural grass field: Bermudagrass (Dry & Wet) | Ta max: 33.3°C | Ambient temperature  Wind velocity  Solar radiation | Young model 05305 anemometer  Campbell Scientific CNR4 net radiometer  Campbell scientific HMP45C temperature and relative humidity probe  Cylindrical radiation thermometer  (1.6 m) |
| (Jim 2016) | 1 x Third-generation synthetic grass field, 35 mm polyethylene pile mono-filament fibres, with 8 mm SBR infill and 10 mm of sand  1 x Natural grass field: Cynodon Dactylon (Bermudagrass) | Ta max: 34.4-34.8°C  Ta min: 28.6-28.9°C  RH mean: 79-90% | Surface temperature  Ambient temperature  Solar radiation | Hobo S-THB thermistor  Apogee SI-11 Infrared radiometer  Lufftt 8160  Hobo S-THB humidity probe  Kipp & Zonen CNR4 net radiometer  (0.15 m, 0.5 m, 1.5 m) |
| (Jim 2017) | 1 x Third-generation synthetic grass field, 35 mm polyethylene pile mono-filament fibres, with 8 mm SBR infill and 10 mm of sand  1 x Natural grass field: Cynodon Dactylon (Bermudagrass). | Sunny day min & max Ta: 28.3 & 34.4°C  Cloudy day min & max Ta: 28.2 & 32.3°C  Overcast day min & max Ta: 27.7 & 30.5°C  Sunny day RH mean: (range): 80% (56-91%)  Cloudy day RH mean (range): 83% (73-88%)  Overcast day RH mean (range): 87% (78-92%) | Surface temperature  Ambient temperature  Solar radiation | Hobo S-THB thermistor  Apogee SI-11 Infrared radiometer  Lufftt 8160  Hobo S-THB humidity probe  Kipp & Zonen CNR4 net radiometer  (Ta & RH: 0.15 m, 0.5 m, 1.5 m)  (Radiation: 1.6 m) |
| (Kandelin et al. 1976) | 1 x Synthetic grass field: Tartan Turf  1 x Natural grass field: Bermudagrass | Solar radiation mean ± SD: 382 ± 22.4 Langley’s  Humidity mean ± SD: 57.3 ± 22.4%  Wind velocity mean ± SD: 7.4 ± 2.7 km/h | Ambient temperature  Surface temperature  Wet-bulb temperature | Tele Tru model 0-150 C and 20-110C) dial and dry-bulb thermometers  Shaded thermometers  Probe thermometers  Cup anemometer  Belfore model 427 Az solar actinometer  (0.9 m & 1.5 m) |
| (Liu and Jim 2021) | 1 x Third-generation synthetic grass field, 35 mm polyethylene pile mono-filament fibres, with 8 mm of SBR and 10 mm of sand infill.  1 x Natural grass field: Bermudagrass | N/a | Surface temperature  Ambient temperature  Solar radiation  Wet-bulb temperature  Relative humidity  Globe temperature  WBGT | Hobo S-THB thermistor  Apogee SI-11 Infrared radiometer  Lufftt 8160  Hobo S-THB humidity probe  Kipp & Zonen CNR4 net radiometer  Ditto  Hobo S-WCA cup anemometer  Hobo S RBG rain gauge  (1.5 m) |
| (Loveday et al. 2019a) | 1 x Synthetic grass plot: Tuff Turf  1 x Natural grass plot: Kikuyu | Winter 2016 Ta range: 0.6-16.5°C  Winter 2016 RH range: 28-98%  Winter 2016 wind velocity mean ± SD: 5.76 ±1.68 m**^.^**s^-1^ | Apparent Temperature | Test 876 thermal imaging camera  Middleton SK010 silicon pyranometer  Rotronic 101A temperature & humidity probe  Synchrotac 706 propeller anemometer  (Surface temperature: 1 m)  (Weather station: 2 m)  (WS: 10 m) |
| (Loveday et al. 2019b) | 1 x Synthetic grass plot: Tuff Turf  1 x Natural grass plot: Kikuyu | Ta max: 27.8 & 41.7°C  Ta min: 23.1& 29.6°C  RH max: 13 & 80%  RH min: 18 & 54% | Solar radiation | Thermochron iButton  (0.02 m) |
| (McNitt et al. 2008) | 1 x Astroplay  1 x Astroturf  1 x Experimental  1 x Field turf  1 x Geoturf  1 x Nexturf  1 x Omnigrass 41  1 x Omnigrass 51  1 x Sofsport  1 x Sprinturf | Ta mean: 25-30°C  RH mean: 33-39%  Wind velocity mean: 1-6 km/h | Surface temperature | Scheduler Model 2 LiCor Corporation infrared thermometer  (1 m) |
| (Petrass et al. 2014a) | 34 x Synthetic grass plots | Ta mean ± SD: 30.25 ± 3.11  RH mean ± SD: 30.28 ± 7.02%  Solar radiation mean ± SD: 698.10 ± 230.89 W**^.^**m^2^  Wind velocity mean ± SD: 5.50 m**^.^**s^-1^ ± 3.69 m**^.^**s^-1^ | Surface temperature | Extech Model HD 500 multimeter with a psychrometer and infrared gauge  Digitech Model QM1642 anemometer  Apogee MP-200 pyranometer  (1 m) |
| (Petrass et al. 2014b) | 1 x Third-generation synthetic grass field, 42 mm polyethylene fibres and crumb rubber infill  1 x Third-generation synthetic grass field, 36mm cool climate polyethylene fibre with crumb rubber.  1 x Natural grass field: Rye grass  1 x Natural grass field: 50% Poa and Rye and 50% kikuyu irrigated weekly | Ta range: 14.4-36.1°C | Surface temperature  Ambient temperature  Wet-bulb temperature  Relative humidity  Wind velocity | Extech Model HD 500 multimeter with a psychrometer and infrared gauge  Digitech Model QM1642 anemometer  (1 m) |
| (Pryor et al. 2017) | 1 x Third-generation synthetic grass field, with SBR  1 x Synthetic grass field: Astroturf  1 x Natural grass field | Ta: 22.5-35.6°C | WBGT | Krestel 4600 heat stress tracker  (1.2 m) |
| (Ramsey 1982) | 1 x Synthetic grass field: Astroturf  1 x Natural grass field: Bermudagrass | N/a | Ambient temperature  Wet-bulb temperature  Globe temperature  WBGT | Reuter Stokes Model 211A heat stress meter  (0.4 m) |
| (Shi and Jim 2022) | 1 x Third-generation synthetic grass field, 35mm polyethylene mono-filament fibres, 8 mm of SBR and 10mm of sand, with a 25mm shock pad  1 x Natural grass field: Bermudagrass | Ta max: 30.5-34.8°C  Ta min: 25.3-28.9°C  RH mean: 76-89% | Surface temperature  Ambient temperature  Wind velocity  Mean radiant temperature | Hobo S-THB thermistor  Apogee SI-11 Infrared radiometer  Lufftt 8160  Hobo S-THB humidity probe  Kipp & Zonen CNR4 net radiometer  Ditto  Hobo S-WCA cup anemometer  Hobo S RBG rain gauge  (1.5 m) |
| (Thoms et al. 2014) | 10 x Synthetic grass plots  Monofilament, pile height 5.1cm, polyethylene and nylon  Monofilament, pile height 3.2 cm nylon  Monofilament, pile height 5.1 cm polyethylene and nylon  Monofilament, pile height 5.7 cm polyethylene  Monofilament. pile height 5.1 cm exp polyethylene and nylon  Slit film, pile height 5.7 cm polyethylene  Monofilament, pile height 5.7 cm exp polyethylene  Monofilament, pile height 5.7 polyethylene  Monofilament pile height 5.7 cm polyethylene  Monofilament/slit film. Pile height 5.1 cm exp polyethylene and nylon  Slit film, pile height 5.1 cm exp polyethylene and nylon | N/a | Surface temperature | TiDbiT v2 temperature logger  HOBO U30/  (Height N/a) |
| (Twomey et al. 2016) | 1 x Third-generation synthetic grass field, 12mm shock pad, 42 mm polyethylene fibre, with sand and recycled rubber particles.  1 x Artificial Street soccer field, 19mm polyethylene fibres with siliceous sand infill  1 x Natural grass field: Rye grass | Ta mean ± SD: 25.6°C ± 5.9°C  RH mean ± SD: 41.3 ± 12.0%.  Wind velocity mean ± SD: 7.6 km/h ± 4.8 km/h | Surface temperature | Extech Model HD 500 multimeter with a psychrometer and infrared gauge  (1 m) |
| (Villacañas et al. 2017) | 14 x Third-generation synthetic grass fields | Ta mean ± SD: 33.67 ± 2.36°C  RH mean ± SD: 22.03 ± 3.11% | Surface temperature | FLIR T420bx infrared thermometer  (0.7 m) |
| (Wardenaar et al. 2022) | 1 x Synthetic grass field, HydroChill turf fibres  1 x Indoor synthetic grass field  1 x Natural grass field | N/a | Surface temperature  Ambient temperature  Relative humidity  Globe temperature  Vapor pressure  WBGT  Wind velocity  Solar radiation | Arable weather station  Krestel 5400 heat stress meter  DeltaTrak infrared thermometer  FLIR T4340sc infrared camera  (1.2 m) |
| (Xiao and Cao 2013) | 1 x Artificial turf lawn  1 x Natural grass lawn | N/a | Surface temperature  Ambient temperature  Relative humidity | Testo self-recording instrument  Infrared thermometer  (0.1 m, 1.0 m, 1.5 m, 2.0 m) |

Key: WBGT (wet-bulb-globe temperature), Ta (ambient temperature), RH (relative humidity), max (maximum), and min (minimum).
