# Supplementary material for: The effect of synthetic grass sports surfaces on the thermal environment: A systematic review
Source: Int J Biometeorol. 2024 May 1;68(7):1235–52. doi: 10.1007/s00484-024-02679-5 (PMC11272752; doi:10.1007/s00484-024-02679-5)
Supplement: Supplementary file 3 — (DOCX 18 kb) [file 484_2024_2679_MOESM3_ESM.docx]

## Article title: The Effect of Synthetic Grass Sports Surfaces on the Thermal Environment: A Systematic Review

**Journal name:** International Journal of Biometeorology
**Author names:** Gurpreet Singh^1^, Benjamin Peterson^2^, Ollie Jay^3^, Christopher. J. Stevens^1^

**Affiliations:** ^1^Physical Activity, Sport, and Exercise Research Theme, Faculty of Health, Southern Cross University, Coffs Harbour, NSW, Australia

^2^School of Health, Medical and Applied Sciences, Central Queensland University, Australia

^3^Heat and Health Research Incubator, Faculty of Medicine and Health, University of Sydney, Australia

**Email:** Gurpreetsinghphd1@gmail.com

**Supplementary File 3.** *A summary of the quality appraisal results*

| Study | Is the study relevant to the needs of the project? | Does the paper address a clearly focused issue? | Is the choice of study method appropriate? | Are the surfaces studied appropriately? | Is confounding and bias considered? | Are tables/graphs adequately labelled and understandable? | Are you confident with the authors' choice and use of statistical methods, if employed? | Can the results be applied to the local situation? | Were all important outcomes/results considered? | Accept for further use as Type IV evidence |
| --- | --- | --- | --- | --- | --- | --- | --- | --- | --- | --- |
| (Bozdogan Sert et al. 2021) | Yes | Yes | Yes | No | Can't tell | Yes | Not employed | Yes | No | Yes |
| (Carvalho et al. 2021) | Yes | Yes | Yes | No | Yes | Yes | Not employed | Yes | Yes | Yes |
| (Grundstein and Cooper 2020) | Yes | Yes | Yes | Yes | Yes | No | No | Yes | Yes | Yes |
| (Guyer et al. 2021) | Yes | Yes | Yes | Yes | Yes | Yes | Yes | Yes | Yes | Yes |
| (Hardin and Vanos 2018) | Yes | Yes | Yes | Yes | Yes | Yes | Yes | Yes | Yes | Yes |
| (Jim 2017) | Yes | Yes | Yes | Yes | Yes | Yes | Not employed | Yes | Yes | Yes |
| (Jim 2016) | Yes | Yes | Yes | Yes | Yes | Yes | Not employed | Yes | No | Yes |
| (Kandelin et al. 1976) | Yes | Yes | Yes | Yes | Yes | Yes | Yes | Yes | Yes | Yes |
| (Liu and Jim 2021) | Yes | Yes | Yes | Yes | Yes | Yes | Not employed | Yes | No | Yes |
| (Loveday et al. 2019a) | Yes | Yes | Yes | Yes | Yes | Yes | Not employed | No | Yes | Yes |
| (Loveday et al. 2019b) | Yes | Yes | Yes | No | Yes | Yes | Not employed | Can't tell | Yes | Yes |
| (McNitt et al. 2008) | Yes | Yes | Yes | Yes | Yes | Yes | Not employed | Can't tell | Yes | Yes |
| (Petrass et al. 2014a) | Yes | Yes | Yes | Yes | Yes | Yes | Yes | Yes | Yes | Yes |
| (Petrass et al. 2014b) | Yes | Yes | Yes | Yes | Yes | Yes | Yes | Yes | Yes | Yes |
| (Pfautsch et al. 2022) | Yes | Yes | Yes | Yes | Yes | Yes | Yes | Can’t tell | Yes | Yes |
| (Pryor et al. 2017) | Yes | Yes | Yes | Yes | Can’t  tell | Yes | No | Can't tell | Yes | Yes |
| (Ramsey 1982) | Yes | Yes | Yes | Yes | Yes | No | Not employed | Yes | Yes | Yes |
| (Shi and Jim 2022) | Yes | Yes | Yes | Yes | Yes | Yes | Not employed | Yes | No | Yes |
| (Thoms et al. 2014) | Yes | Yes | Yes | Yes | Yes | Yes | Yes | Can't tell | Yes | Yes |
| (Twomey et al. 2016) | Yes | Yes | Yes | Yes | Yes | Yes | Yes | Yes | Yes | Yes |
| (Villacañas et al. 2017) | Yes | Yes | Yes | Yes | Yes | Yes | Yes | Yes | Yes | Yes |
| (Wardenaar et al. 2022) | Yes | Yes | No | Yes | No | Yes | Not employed | Yes | Yes | Yes |
| (Xiao and Cao 2013) | Yes | Yes | Yes | Yes | Can’t tell | Yes | Not employed | Yes | Yes | Yes |

Key. Not employed (not available was applied when the study did not make statistical comparisons).
